# Resonant Cholinergic Dynamics in Cognitive and Motor Decision-Making: Attention, Category Learning, and Choice in Neocortex, Superior Colliculus, and Optic Tectum

**DOI:** 10.3389/fnins.2015.00501

**Published:** 2016-01-20

**Authors:** Stephen Grossberg, Jesse Palma, Massimiliano Versace

**Affiliations:** ^1^Graduate Program in Cognitive and Neural Systems, Boston UniversityBoston, MA, USA; ^2^Center for Adaptive Systems, Boston UniversityBoston, MA, USA; ^3^Departments of Mathematics, Psychology, and Biomedical Engineering, Boston UniversityBoston, MA, USA; ^4^Center for Computational Neuroscience and Neural Technology, Boston UniversityBoston, MA, USA

**Keywords:** category learning, saccadic eye movement, attention, adaptive resonance theory, superior colliculus, optic tectum, acetylcholine, vigilance

## Abstract

Freely behaving organisms need to rapidly calibrate their perceptual, cognitive, and motor decisions based on continuously changing environmental conditions. These plastic changes include sharpening or broadening of cognitive and motor attention and learning to match the behavioral demands that are imposed by changing environmental statistics. This article proposes that a shared circuit design for such flexible decision-making is used in specific cognitive and motor circuits, and that both types of circuits use acetylcholine to modulate choice selectivity. Such task-sensitive control is proposed to control thalamocortical choice of the critical features that are cognitively attended and that are incorporated through learning into prototypes of visual recognition categories. A cholinergically-modulated process of vigilance control determines if a recognition category and its attended features are abstract (low vigilance) or concrete (high vigilance). Homologous neural mechanisms of cholinergic modulation are proposed to focus attention and learn a multimodal map within the deeper layers of superior colliculus. This map enables visual, auditory, and planned movement commands to compete for attention, leading to selection of a winning position that controls where the next saccadic eye movement will go. Such map learning may be viewed as a kind of attentive motor category learning. The article hereby explicates a link between attention, learning, and cholinergic modulation during decision making within both cognitive and motor systems. Homologs between the mammalian superior colliculus and the avian optic tectum lead to predictions about how multimodal map learning may occur in the mammalian and avian brain and how such learning may be modulated by acetycholine.

## 1. Attention, learning, and vigilance during cognitive category learning in temporal cortex

Selecting relevant sensory information while interacting with a changing environment is a key feature of animal intelligence. This selection is necessary to direct limited sensory, cognitive, and motor resources toward the important stimuli in the environment, and to choose a set of motor commands that correspond to behavioral goals. The present article proposes how cholinergic modulation of cognitive and sensory-motor circuits may realize such selectivity in a task-sensitive way. In particular, a shared circuit design in cognitive and sensory-motor circuits is proposed to enable acetylcholine to effectively modulate selectivity during decision-making via a process called *vigilance control* (Carpenter and Grossberg, 1987, 1991, 1993). High vigilance implies greater selectivity, whereas low vigilance implies lesser selectivity. The proposal of how vigilance may regulate the degree of selectivity during cognitive and motor decision-making builds upon two parallel lines of neural modeling whose results are unified and extended in the current article.

One line of modeling developed the LAMINART model of how the laminar circuits of visual cortex see and learn visual recognition categories (e.g., Grossberg, 1999, 2003; Grossberg and Raizada, 2000; Raizada and Grossberg, 2001). The second line of modeling developed the SACCART model of how the mammalian superior colliculus learns a multimodal map wherein saccadic target positions can be attended and chosen. Both of these modeling streams illustrate how Adaptive Resonance Theory, or ART, design principles and mechanisms are used to learn recognition categories. The current article unifies both modeling streams into a more general theory of how brain categories are learned and used to control visual and sensory-motor behaviors.

Several key steps in this unification are developed herein. One step began with the proposal of a further development of the LAMINART model, namely the Synchronous Matching ART or SMART model (Grossberg and Versace, 2008). As noted above, ART had earlier predicted how the selectivity, notably the concreteness or abstractness, of learned visual cortical categories is controlled by a process of vigilance control. SMART further developed this proposal by suggesting that vigilance may be controlled by mismatch-activated release of acetylcholine via the nucleus basalis of Meynert. The current article describes how these results about visual cortical categories may be adapted to explain the selectivity of learning and choice by sensory-motor categories. This theme is developed by noting homologs between the mammalian superior colliculus and the avian optic tectum in the control of eye movements. It is shown that the key predictions of the LAMINART, SMART, and SACCART models are supported by a series of experiments on the optic tectum. In particular, a refinement of the SACCART model anatomy enables a detailed explanation of many optic tectum data as embodiments of LAMINART, SMART, and SACCART design principles and mechanisms. The theory developed herein also makes new predictions about sensory-motor categories and their dynamics in superior colliulus and optic tectum for which no data seem to be currently available.

Each of these lines of model development about cognitive and sensory-motor processing has been supported by mathematical theorems and/or computer simulations that have quantitatively explained and predicted challenging psychological and neurobiological data, as well as rigorously demonstrated key model properties. This foundation of prior modeling results provides a secure foundation for the theoretical synthesis that is provided in the current article, without requiring additional simulations to justify theoretical claims.

In models of how cognitive recognition categories are learned and recalled (Carpenter and Grossberg, 1987, 1991, 1993; Grossberg, 2013a), low vigilance leads to learning of a general, or abstract, recognition category, whereas high vigilance leads to learning of a specific, or concrete, recognition category. In the limit of very high vigilance, such a category may learn to represent a single input exemplar, such as a particular view of a particular familiar face. Such learning is proposed to occur in both bottom-up and top-down thalamocortical and corticocortical pathways, notably the temporal cortex and its interactions with prefrontal cortex and the thalamus. The bottom-up learning helps to select a recognition category, whereas the top-down learning enables read-out of learned top-down expectations that can focus attention upon expected combinations of critical features. The critical features that are learned under high vigilance can only be matched by very similar input exemplars, thereby controlling a highly specific attentional focus, whereas the critical features that are learned under low vigilance can be matched by much more variable combinations of features, thereby controlling a broader distribution of objects that can be assimilated into the attentional focus. Top-down expectation mechanisms achieve such attentional and choice properties via connections that are organized as recurrent on-center, off-surround networks (Grossberg, 2013b). The on-center helps to select and amplify consistent features that are received within the attentional focus, while the off-surround suppresses unattended features or positions outside this focus. Models of this kind are called Adaptive Resonance Theory or ART models (Grossberg, 1980, 1999, 2007, 2013a; Carpenter and Grossberg, 1987, 1991).

ART proposes a solution of the *stability-plasticity dilemma*, or how brains can learn quickly without also catastrophically forgetting already learned memories just as quickly (Grossberg, 1980). ART explains how top-down attentive matching may help to solve the stability-plasticity dilemma by regulating cycles of *resonance* and *reset*; that is, of attentive matching and hypothesis testing, respectively. In particular, when a good enough match occurs between bottom-up inputs and a top-down expectation, then a synchronous resonant state emerges that embodies an attentional focus that is capable of driving fast learning of the attended critical features in both bottom-up recognition categories and top-down expectations; hence the name *adaptive* resonance. If the match is not good enough, then the currently active recognition category is reset by a complementary orienting system, and interactions between the attentional and orienting systems drive a search for a new or better-matching category.

All the key predictions of ART, including those about vigilance control, have received support from psychological and neurobiological experiments. See below and reviews by Grossberg (1999, 2003, 2013a,b), Grossberg and Versace (2008), and Raizada and Grossberg (2003). The potential significance of the vigilance concept is illustrated by the prediction that various autistic individuals may have their vigilance stuck at abnormally high levels, thereby helping to explain the hyper-concreteness of autistic attention and learning (Grossberg and Seidman, 2006; Church et al., 2010; Vladusich et al., 2010). Grossberg and Versace (2008) developed the Synchronous Matching ART, or SMART, model to explain how laminar circuits in visual cortex whose cells obey spiking dynamics can carry out visual category learning. SMART additionally predicted how vigilance in these laminar cortical circuits may be regulated by acetycholine (ACh) via the nucleus basalis of Meynert. Consistent with this proposal are data about autistic individuals showing abnormal ACh activity in the parietal and frontal cortices that is correlated with abnormalities in the nucleus basalis (Perry et al., 2001; Ray et al., 2005).

## 2. Attention, learning, and vigilance during motor category learning in superior colliculus

Another circuit that seems to embody ART dynamics has been proposed to exist in the deeper layers of the superior colliculus (SC). The SACCART model (Grossberg et al., 1997) proposes how a multimodal map that attentively selects saccadic eye movement target positions may be learned within the deeper layers of the SC. Unimodal inputs to the SC come from several different brain regions, including auditory, visual, and prefrontal cortical areas. Learning combines all of these inputs into a multimodal map for saccadic choice. Learning routes the SC connections of these auditory, visual, and prefrontal planning inputs so that all these inputs can activate the same target positions, despite their different inputs sources, using—as in the case of cognitive category attention, choice, and learning—a recurrent on-center off-surround network as a choice network. In the SC, these learned connections enable any combination of auditory, visual, and cognitive input sources to compete within the deeper SC layers to select the target position of the next saccade. These interactions enable the model to quantitatively simulate the temporal dynamics of SC burst and buildup cells under a variety of experimental conditions. Burst cells respond with bursts that decay as the next saccadic position is chosen and executed. Buildup cells generate a spatially distributed pattern of activity that begins at the chosen position and then spreads toward the position of the fovea as the chosen saccadic command causes the eye to foveate. Because these dynamics are modeled by a specialized ART circuit, this motor map learning process may be viewed as a kind of attentive motor category learning.

The current article proposes that, just as in the case of cognitive category learning, the SC circuit for motor category learning uses ACh to sharpen the map loci that make saccadic choices, and does so in a manner similar to the way that ACh may modulate the vigilance of cognitive category choice and learning. Recent neurophysiological results about the avian equivalent of the SC, the optic tectum (OT), are consistent with the SACCART model. The OT data also have the advantage that they include the results of an ACh manipulation that is consistent with this ART prediction. Thus, the SC and OT may both be useful experimental models for studying vigilance control during attentive motor category learning. The current article reviews key data about the anatomy and neurophysiology of OT to set the stage for explaining how these OT data support ART predictions about motor category learning under ACh-modulated vigilance control.

## 3. Cholinergic modulation of attention and choice in the optic tectum

Indeed, in pigeons, a topographically organized ACh signal to the OT is part of a midbrain neural circuit that helps to choose and pay attention to one visual stimulus from among the many stimuli that occur within their view. Whenever a visual stimulus activates OT neurons in a given tectal position, this position receives strong bursting feedback from ACh neurons of the nucleus isthmi pars parvocellularis (Ipc) that is located under the tectum. If a second visual stimulus is presented, the feedback signal to the first tectal position is reduced or suppressed, while feedback to the second tectal position begins. This long-range inhibition is received primarily from the nucleus isthmi pars magnocellularis (Imc), which sends a broad GABAergic projection to the Ipc and OT.

At least two types of data support the idea that feedback from the Ipc modulates OT output: First, the thalamic nucleus rotundus (RtDa), which receives the ascending tectal output, exhibits visually evoked extracellular responses that are synchronized to this feedback signal. Second, if the Ipc is inactivated, then visual responses in RtDa are prevented in response to visual targets that move in the corresponding region of space. In summary, the ascending transmission of visual activity is gated by this ACh feedback signal, whose position within the OT visual map is dynamically controlled by competitive interactions (Wang, 2003; Wang et al., 2004; Marín et al., 2007).

These feedback interactions cause oscillatory bursts and switch-like properties that rapidly increase cell responses to the strongest stimulus in their receptive field (Marín et al., 2005; Asadollahi et al., 2010), both properties of ART resonance and reset, respectively. As in the SC, there is multimodal fusion of auditory and visual inputs in the OT-Ipc network (Maczko et al., 2006), consistent with multimodal map learning of the kind modeled by SACCART.

The remainder of this article reviews and refines properties of cognitive and motor category learning by ART models, and also uses these theoretical results to explain how OT dynamics illustrate ART mechanisms for map learning and choice. These theoretical connections thereby explicate OT dynamics and facilitate use of the OT as a paradigm for further investigating motor category learning and ACh-modulated vigilance control.

## 4. Adaptive resonance theory

### 4.1. Attention, resonance, and stable category learning

A comprehensive heuristic review of ART is given in Grossberg (2013a). Here are reviewed those properties that are needed to build the bridge between cognitive and motor category learning and ACh modulation that is the primary focus of the present article.

Humans and other primates are *intentional* beings: they learn expectations and make predictions about what is about to happen in the world. Humans are also *attentional* beings: they restrict processing resources to a limited amount of incoming information at any time. Why do humans and other primates carry out both intentional and attentional processing? How are these processes related? The stability-plasticity dilemma and its solution using resonant states provides a unified answer.

The role of sensory or cognitive expectations, and of how a resonant state is activated, are illustrated by the following task: “find the blue glass as quickly as possible, and you will win a $10,000 prize.” When an expectation of a “blue glass” is active, the glass can be more rapidly and energetically detected. Thus, sensory and cognitive top-down expectations are realized by a process of *excitatory matching* with consistent bottom-up data. When a mismatch occurs between top-down expectations and bottom-up data, it suppresses the mismatched features of the bottom-up data, so that attention can be focused upon the matched, or expected, features.

A good enough match between bottom-up and top-down signal patterns between two or more levels of processing generates a resonant state in which their positive feedback signals amplify, prolong, and synchronize the mutual activation between the attended features and their category. Resonance triggers learning in the more slowly varying adaptive weights that control the signal flow along pathways from cell to cell. Resonance is thus a global context-sensitive state that supports data worthy of learning, hence the name *Adaptive* Resonance Theory.

In summary, ART unifies brain mechanisms that enable advanced brains to quickly and stably categorize information about currently active feature patterns using bottom-up pathways, with mechanisms that enable expectations to be learned about these feature patterns using top-down pathways. Read-out of such a top-down expectation “tests a hypothesis” that the currently active category is a sufficiently good representation of the bottom-up feature pattern that is also then active. When a sufficiently good match occurs between the currently active bottom-up feature pattern and the learned top-down expectation, then resonance can be triggered and focus attention upon this critical features that are read-out by the expectation. By learning only attended features, ART clarifies how, in order to solve the stability-plasticity dilemma, only resonant states can drive rapid new learning.

ART furthermore predicts that “all conscious states are resonant states.” This prediction has been supported by many modeling studies whose computer simulations of behavioral and brain data using resonant states provide a linking hypothesis between brain dynamics and conscious experiences. That is, emergent properties of resonant states map onto parametric properties of conscious experiences in the simulated experiments.

The type of learning within the sensory, cognitive, and motor domain that ART mechanizes is *match learning*: Match learning is so called because it occurs only if a good enough match occurs between bottom-up patterns and learned top-down expectations that are read out by a currently active recognition category. A good enough match enables previously learned knowledge to be refined.

### 4.2. Complementary computing: resonance and reset

Carpenter and Grossberg (1987, 1991) have mathematically proved that match learning within an ART model leads to stable category memories in response to arbitrary lists of events. However, match learning is insufficient by itself to learn from a changing world. Indeed, if the brain can only rapidly learn when there is a good enough match between bottom-up data and learned top-down expectations, then how does the brain ever learn anything that is truly novel? ART shows how this problem may be solved using interactions between complementary processes of *resonance* and *reset*. Resonance controls properties of attention and learning that have already been discussed. Reset controls properties of hypothesis testing and memory search that will be discussed now. Working together, these complementary processes enable our brains to balance between the complementary demands of processing familiar vs. unfamiliar information, and expected vs. unexpected information.

The resonance process during visual category learning takes place in the What cortical stream, notably the inferotemporal and prefrontal cortex. As discussed above, it is here that top-down expectations are matched against bottom-up feature patterns. When a good enough match occurs, it focuses attention upon the features in the bottom-up feature pattern that are expected. If the expected pattern is close enough to the input pattern, then a state of resonance develops as attention focuses on the expected subset of features.

**Figure 1:**
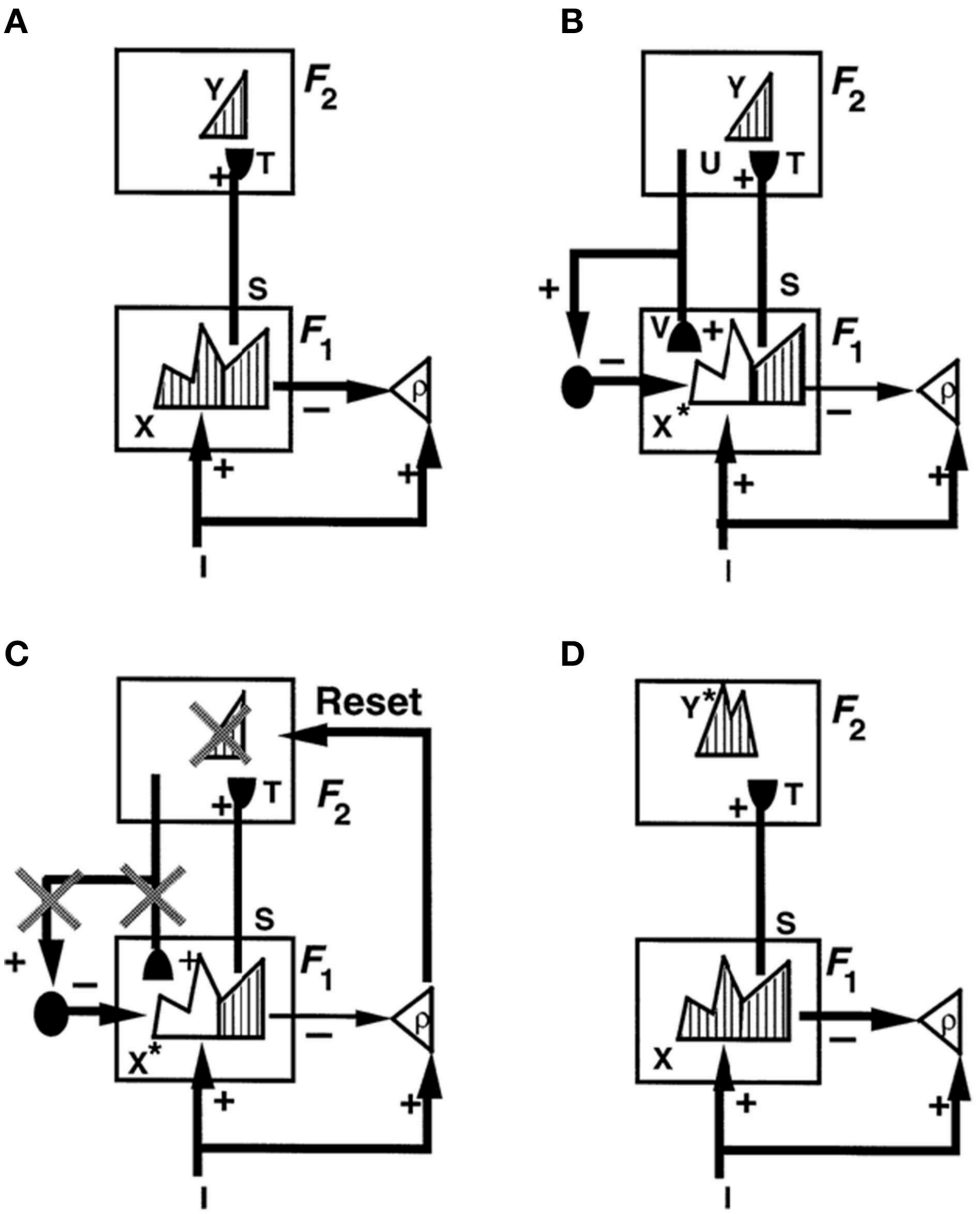
**How ART searches for a recognition category using cycles of resonance and reset. (A)** Input pattern *I* is instated across feature detectors at level *F*_1_ as an activity pattern *X*, while it non-specifically activates the orienting system *A* with gain ρ, the *vigilance* parameter. *X* inhibits *A* and generates output pattern *S. S* is multiplied by learned adaptive weights to form the input pattern *T. T* is contrast-enhanced and normalized by recurrent shunting competition, leading to selection and activation of category cells *Y* at level *F*_2_. **(B)** The category activity *Y* generates the top-down signals *U* which are multiplied by adaptive weights to form a *prototype V* that encodes the learned expectation of active *F*_2_ categories. The top-down expectation *V* is added at *F*_1_ cells. If *V* mismatches *I* at *F*_1_, then a new STM activity pattern X^*^ (the hatched pattern) at cells where the patterns match sufficiently is selected at *F*_1_. X^*^ is active at *I* features that are confirmed by *V*. Mismatched features (white area) are inhibited. When *X* changes to X^*^, total inhibition decreases from *F*_1_ to *A*. **(C)** If inhibition decreases sufficiently, *A r*eleases a non-specific arousal burst to *F*_2_; that is, “novel events are arousing.” Arousal resets *F*_2_ by inhibiting *Y*. **(D)** After *Y* is inhibited, *X* is reinstated and *Y* stays inhibited for a while as *X* activates a different activity pattern Y^*^. Search continues until a better matching or novel category is selected. When search ends, an attentive resonance triggers learning of the attended data. Adapted with permission from Carpenter and Grossberg (1993).

Figure 1 illustrates these ART ideas in a simple example. In Figure 1A, a bottom-up input pattern, or vector, *I* activates a pattern *X* of activity across the feature detectors of the first level *F*_1_. For example, a visual scene may be represented by boundary and surface features. The differences in the activity pattern *X* represent the relative importance of different features in the input pattern *I*. In Figure 1A, pattern peaks represent more activate feature cells, and troughs less activate feature cells. Activity pattern *X* triggers signal pattern *S* within the bottom-up connections of an adaptive filter to the second level *F*_2_. Before *S* can reach level *F*_2_, each signal in *S* is multiplied by an adaptive weight, or long-term memory trace, thereby giving rise to the input vector *T* to *F*_2_. Each adaptive weight can be altered through learning. When *T* inputs to *F*_2_, it activates a compressed representation, category, or symbol *Y* in response to the more distributed input *T*. Representation *Y* is compressed by competitive, or lateral inhibitory, interactions across *F*_2_ that select a small subset of the most strongly activated cells, while inhibiting cells that receive smaller inputs. The pattern *Y* in Figure 1A is drawn to illustrate that the small number of category cells may be activated to different degrees. The active category cells *Y* can then send top-down signals *U* back to *F*_1_. The vector *U* becomes a top-down expectation *V* when it is multiplied by a matrix of top-down adaptive weights. Matching across *F*_1_ occurs between the bottom-up input vector *I* and the top-down expectation *V*. Matching selects the subset X^*^ of features within *X* that are confirmed by *V*. These selected features constitute the “attentional focus.”

### 4.3. Binding distributed feature patterns and symbols during a conscious resonance

If the top-down expectation *V* is similar enough to the bottom-up input pattern *I*, then the pattern X^*^ of attended features can reactivate category *Y*. Category *Y*, in turn, reactivates X^*^. This positive feedback cycle leads to a synchronous resonant state that can enter consciousness.

This coherent state provides a solution of the classical “symbol grounding problem” (Harnad, 1990). The two levels *F*_1_ and *F*_2_ experience complementary types of ignorance: Activating a category at *F*_2_ can represent a distributed feature pattern, but the category has no information about what these features are. Activating a feature detector at *F*_1_ does provide such information, but individual features have no meaning by themselves. The resonant bound state binds the *pattern* of critical features to the category that represents them.

A resonance can generate either a stable equilibrium or a synchronous oscillation. The article that introduced ART (Grossberg, 1976b) predicted the existence of such synchronous oscillations. They were called “order-preserving limit cycles” because they preserve the ordering of activities as they synchronously oscillate through time. In contrast, order-reversing oscillations could, for example, support a traveling wave or epileptic seizure. Grossberg (2003, 2013a) review psychological and neurobiological data that support all the main ART predictions, including predictions about synchronous oscillations.

### 4.4. Resonance links intention and attention to learning

In ART, the resonant state is predicted to drive learning. Its synchronization, amplification, and prolongation of activity is sufficient to activate slower learning processes in the adaptive weights within the bottom-up and top-down pathways between levels *F*_1_ and *F*_2_ in Figure 1. Adaptive weights that were changed through previous learning can hereby regulate current information processing, without necessarily learning about the signals that they process unless they can initiate a resonant state. Thus, adaptive resonance is a mediating event that solves the stability-plasticity dilemma and, in so doing, provides a mechanistic explanation of why humans are intentional beings who continually predict what may next occur, and why humans tend to learn about events to which they pay attention.

The fact that humans can also sometimes learn without attention or conscious awareness, for example during perceptual learning, is also explained by ART, but how this is proposed to happen goes beyond the scope of this review. See Grossberg (2003, 2013a) for reviews.

### 4.5. Complementary attentional and orienting systems control resonance and reset

When a sufficiently bad mismatch occurs between an active top-down expectation and a bottom-up input that represents an unexpected or unfamiliar event, it can drive a memory search by activating the *orienting system*. The orienting system obeys computationally complementary laws from those of the attentional system that carries out category learning and top-down attentional matching. In particular, the orienting system is activated by unexpected and unfamiliar events. ART proposes that the attentional system includes temporal and prefrontal cortex, whereas the orienting system includes the non-specific thalamus and the hippocampal system, among other brain regions. Output signals from the orienting system rapidly reset the recognition category within the attentional system that read out the poorly matching top-down expectation (Figures 1B,C). The cause of the mismatch is hereby removed. The attentional system can then activate a different recognition category (Figure 1D). The reset event hereby triggers memory search, or hypothesis testing, for a recognition category that better matches the input pattern.

No such recognition category may currently exist if the bottom-up input represents a truly novel experience. In this situation, the search process activates an as yet uncommitted population of cells, with then learn to categorize the novel input pattern. The ability to activate an uncommitted population cannot be taken for granted. It happens within ART because of the way that the category level *F*_2_ is designed. One important property is that the total activity across *F*_2_ tends to be conserved, due to the recurrent shunting on-center off-surround interactions that store chosen categories in short-term memory in *F*_2_ (Grossberg, 1973, 1980). This property helps to compensate for the fact that, after a disconfirmed category is inhibited by reset, the adaptive weights which activate the new category will typically be smaller, or worse matched, than those that activated the inhibited category. Thus, although the inputs to the newly chosen category can only initially activate it less than its predecessor, the normalized total activity can amplify this initial activity to fully activate the newly chosen category.

In addition, the top-down expectation that is activated by a newly chosen recognition category must be able to match whatever input feature pattern caused it to be activated, so that learning can begin. This property is ensured by choosing all top-down adaptive weights to initially have large values. Learning of a top-down expectation thus *prunes* these weights to match the critical feature pattern that is learned by the category's bottom-up adaptive filter.

This learning process works well under both unsupervised and supervised conditions (Carpenter and Grossberg, 1987, 1991; Carpenter et al., 1992, 2005; Amis and Carpenter, 2009). Unsupervised learning means that the system can learn to categorize novel input patterns without an external teacher. Input patterns are categorized together based upon their similarity alone, although how the criterion of acceptable similarity is set, called *vigilance control*, needs to be understood; see Section 5. Supervised learning also uses vigilance control. In addition, when the system predicts an answer, a teaching signal from the environment can match or mismatch this prediction. If the prediction causes a big enough mismatch, this can activate the orienting system and force a memory search for a new category that can learn a better-matching prediction. Supervised learning is often important when the answers to be learned are culturally determined, and are not based on feature similarity alone. For example, separating the featurally similar letters C and O, or E and F, into separate recognition categories is culturally determined. On a learning trial when O is predicted in response to presentation of C, supervised feedback enables the system to learn separate categories and top-down expectations for C and O.

In summary, the complementary processes of attentive-learning and orienting-search can, through their interactions, enable incremental learning and hypothesis testing that together can build a self-refining internal model of a changing world.

### 4.6. Mismatch-mediated arousal, habituative synapses, and reset

How does a reset signal lead to selection of a new category that can better match and predict the world? How does such a search work during unsupervised learning when there is no external teacher? Indeed, how does search work during unsupervised learning despite the fact that, when the mismatch occurs, the correct answer is not known, and the orienting system has no knowledge of which category caused the reset?

This state of affairs illustrates another example of complementary processing by the brain: Within the attentional system, the chosen category is known, but there is no knowledge of whether it is correct enough to support resonance and learning. Within the orienting system, it is known if an error occurred within the attentional system, but not which category caused it. How do the two systems interact to overcome their complementary deficiencies and discover a better-matching, possibly entirely new, category?

A solution to this search problem was proposed by Grossberg (1976b, 1980). This solution predicts that the pathways that mediate reset utilize *habituative transmitter gates*, which are a form of medium-term memory (MTM), distinct from the short-term memory (STM) that describes rapid cell activation, and the long-term memory (LTM) that persists after learning occurs. Laws for habituative gating MTM, as well as of STM and LTM, were introduced in Grossberg (1968, 1969). These MTM gating processes may, in principle, occur either at presynaptic transmitters or postsynaptic receptors. Neurobiological data and supportive modeling were reported by Abbott et al. (1997) for visual cortex and by Tsodyks and Markram (1997) for somatosensory cortex, using the names *synaptic depression* and *dynamic synapses*, respectively.

These gating processes seem to carry out several roles in the brain. During the processing of sensory inputs, they enable individual cells to adapt their responses to the average level of input intensity, and thereby maintain cell sensitivity to changes in input intensity by contrast-normalizing cell responses to time-varying inputs; e.g., Carpenter and Grossberg (1981), Gaudiano and Grossberg (1991), and Grossberg (1972, 1984b). During cortical map development, they prevent perseverative activation of cells, and thereby allow new inputs to learn how to activate new cells; e.g., Grossberg and Seitz (2003) and Olson and Grossberg (1998). During percepts of changing visual inputs, they limit persistent activation of cells after their inputs end, and thereby prevent moving objects from creating smeared percepts across a scene; e.g., Francis and Grossberg (1996) and Francis et al. (1994). During percepts of visual motion, they enable cells to respond to changing inputs with transient responses; e.g., Baloch et al. (1999), Berzhanskaya et al. (2007), and Öğmen (1993). During bistable visual percepts, habituation of the pathways that support one percept can enable a competing percept to become dominant for a while; e.g., Grossberg and Swaminathan (2004), Grossberg and Yazdanbakhsh (2005), Grossberg et al. (2008), and Wilson (2007). During the learning of ART recognition categories, they enable a reset signal from the orienting system to inhibit categories whose top-down expectations mismatch bottom-up input patterns, and thereby enabling search for better-matching categories to continue; e.g., Carpenter and Grossberg (1987, 1991) and Grossberg (1976b, 1980). All of these examples illustrate how the brain can adapt to variable input intensity levels and reset its responses to respond to changing inputs in as unbiased a way as possible.

How does this search process work? As shown in Figure 1C, when there is a big enough mismatch, the orienting system *A* is activated. This activation generates an output burst that is delivered with equal strength to all targeted thalamocortical cells. This is thus a burst of *non-specific arousal*. It is delivered equally, or non-specifically, to all cells because the orienting system does not know what categories read out the expectation that caused the mismatch. Any of them could have been responsible. It is called an arousal signal because it mechanizes the intuition that “novel events are arousing.” This equal signal to all target cells can selectively reset the cells that are responsible for a predictive mismatch. It does so using the MTM property of habituative gates (Grossberg, 1968, 1969). Grossberg (1972, 1980) proved mathematically that a burst of non-specific arousal can selectively shut off currently active cells and boost the activities of cells that were previously activated but partially suppressed. That is, if non-specific arousal boosts the activation of pathways that are habituatively gated, it can drive a selective memory search for a better-matching category. The laminar cortical circuits in which this is predicted to happen will be described in Section 8 after a summary is given of how big a mismatch is needed to trigger a non-specific arousal burst from the orienting system. These laminar cortical circuits also specify the pathways through which top-down attention modulates cell activations.

## 5. Learning exemplars and prototypes: vigilance control

How general is the featural information that is compressed within a recognition category? Some scientists espouse the view that exemplars, or individual experiences, are learned, corresponding to the fact that some memories are specific and concrete. For example, humans and various other primates can recognize particular views of familiar faces. However, if all memories were stored as exemplars of individual experiences, a combinatorial explosion of memory could ensue, leading to unmanageable problems of memory retrieval. An alternative proposal is that humans learn prototypes that represent general and abstract properties of objects and events (Posner and Keele, 1968). For example, most humans can recognize that other humans have faces. How does the brain learn both specific and concrete exemplars and general and abstract prototypes? ART provides an answer to this question that overcomes problems faced by earlier models. It does so using interactions between its complementary attentional and orienting systems.

ART does learn a kind of prototype, but ART prototypes are not merely averages of the exemplars that are classified by a category, as has been assumed in many prototype models. Instead, ART prototypes are *critical feature patterns* upon which learned top-down expectations of the category focus attention. These critical feature patterns are subsets of the features that have activated the corresponding category in the past. The concreteness or generality of the information that is coded by a critical feature pattern is determined by a gain control process that is called *vigilance control* (Carpenter and Grossberg, 1987, 1993). Vigilance can be altered by different kinds of information, including environmental feedback that is triggered by a predictive error, internal volition, or valued reinforcers. Low vigilance permits the learning of general categories with abstract prototypes. High vigilance forces a memory search to occur for a new category when even small mismatches exist between an input exemplar and the category that it initially activates. In the limit of high vigilance, the category prototype may encode an individual exemplar. In this way, ART regulates the generality of a category to match the predictive demands of each environment in which it learns.

Vigilance is computed within the orienting system. For it to do its job, the bottom-up input pattern *I* to *F*_1_ also activates the orienting system (Figure 1A). Here, the total excitation due to *I* is reduced by inhibition from all the active features across *F*_1_. In particular, the total excitatory input to the orienting system is ρ|*I*|, where |*I*| is the total size of the featural input and ρ is the *vigilance parameter*. When no top-down expectation is active, the total activity across the active features in *F*_1_ is *X*. Then the total inhibition of |*X*| is subtracted from ρ|*I*|, The inequality ρ|*I*| − |*X*| ≤ 0 always occurs when no top-down expectation is active because then |*I*| = |*X*|, since the total number of active inputs equals the total number of activated cells in this case, and ρ ≤ 1. When a top-down expectation is active (Figure 1B), then *X* is transformed into X^*^, so that |X^*^| is subtracted within the orienting system. When inequality ρ|*I*| − |X^*^| ≤ 0 holds, it signifies that the match between the bottom-up input pattern *I* and the learned top-down expectation is good enough to keep the orienting system inhibited. The active category can therefore resonate with the attended features X^*^ to drive new learning.

The inequality ρ|*I*| − |X^*^| > 0 holds when the input pattern *I* is so poorly matched, and thus novel, to require new learning of a different category with which to adequately represent it. The orienting system is then activated and triggers a non-specific reset wave, or arousal burst (Figure 1C). This arousal burst initiates a memory search for a different category with which to classify the exemplar.

The vigilance parameter hereby controls how bad a match will be tolerated before search for a new category is initiated. If vigilance is low, it is easier to prevent an arousal burst from occurring. Under these circumstances, many exemplars can resonate with the same category, leading to learning of a general and abstract prototype that is represented in all these exemplars. In contrast, if vigilance is high, then relatively small differences between a new exemplar and the prototype that was learned in response to the first exemplar can activate the orienting system. In particular, a small difference between a new exemplar, such as O, and a previously learned prototype, such as for C, can drive search for a new category with which to represent O.

When a given environment contains both specific and general information, a fixed value of vigilance may not be sufficient to eliminate all predictive errors. To overcome this problem, vigilance may vary through time to realize *match tracking*. Then, in response to a predictive error, the vigilance parameter ρ increases until it is just big enough to make ρ|*I*| − |X^*^| positive, and thus to drive a memory search for another category (Figures 1C,D). For example, if the letter O activates the category for the previously learned letter C, the network may erroneously predict C. This predictive error can increase vigilance just enough to drive a search for a new category with which to represent O.

Match tracking thus works by making ρ just big enough to exceed the ratio $\frac{\left|X^{*}\right|}{\left|I\right|}$ of the number |X^*^| of active features in *F*_1_ to total features |*I*| in the input pattern *I*. In other words, vigilance then “tracks” the degree of match between input exemplar and matched prototype. By just exceeding the minimal level of vigilance that can trigger a memory search for a new category, match tracking acts like a Minimax Learning Rule: It conjointly *maximizes* category generality as it *minimizes* predictive error. In so doing, match tracking uses the fewest memory resources that are needed to overcome predictive errors. This property clarifies how, for example, children tend to overgeneralize.

## 6. Vigilance control by acetycholine via nucleus basalis during visual category learning

**Figure 2:**
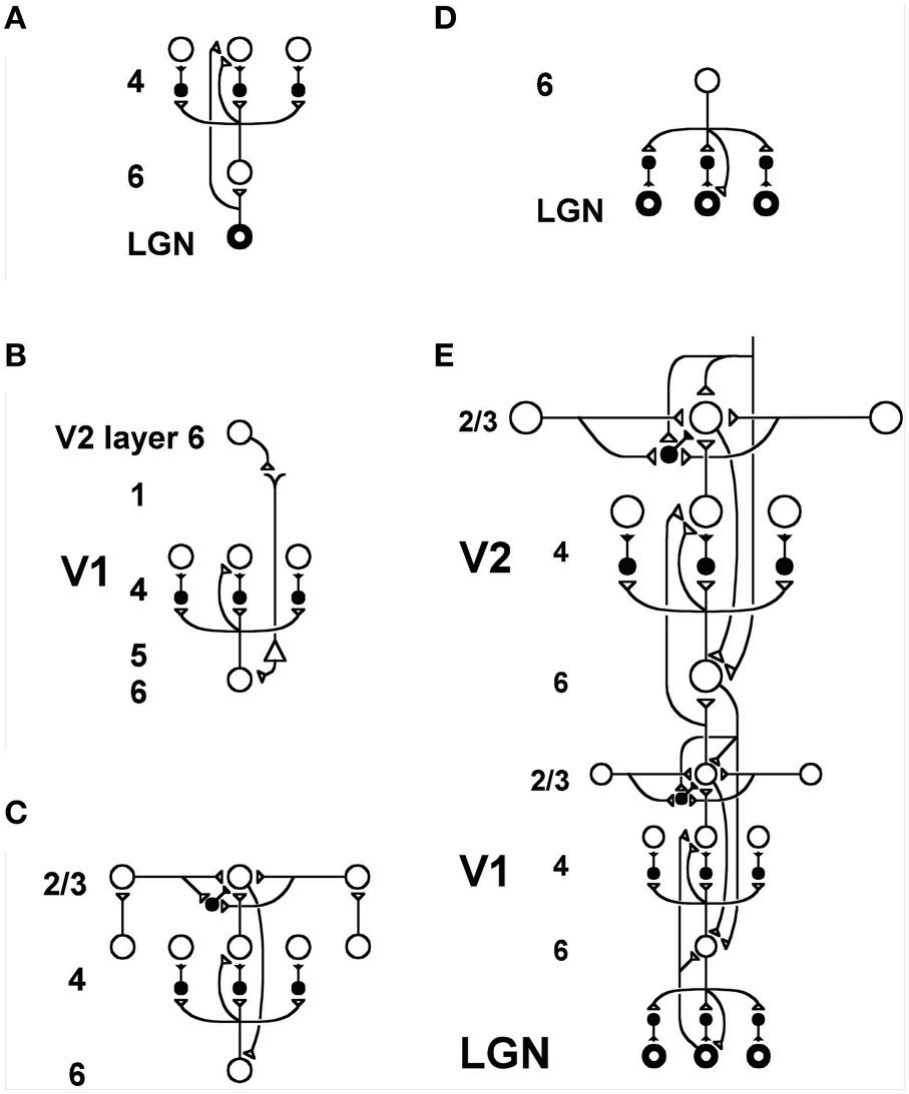
**Model LAMINART circuitry for perceptual grouping and attention in cortical areas V1 and V2**. Inhibitory interneurons are shown filled-in black. **(A)** Two bottom-up input pathways from the lateral geniculate nucleus (LGN) to layer 4 of V1. A strong driving connection goes directly from LGN to layer 4. LGN axons send collaterals into layer 6, and thereby also activate layer 4 via a layer 6 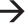 4 modulatory on-center, off-surround network. The combined effect of the bottom-up LGN pathways is to drive layer 4 via an on-center off-surround network which also divisively contrast-normalizes the input pattern (Grossberg, 1973; Heeger, 1992). **(B)** How attention from a higher cortical area reaches layer 4 of a lower cortical area: corticocortical feedback axons tend to originate in layer 6 of the higher area and terminate in layer 1 of the lower cortex, where they can excite apical dendrites of layer 5 pyramidal cells whose axons send collaterals into layer 6. The triangle in the figure represents such a layer 5 pyramidal cell. Several other routes through which feedback can pass into V1 layer 6 exist. Having arrived in layer 6, the feedback is then “folded” back up into the feedforward stream by passing through the 6 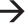 4 on-center off-surround path. This circuit realizes the top-down, modulatory on-center, off-surround circuit of the ART Matching Rule. **(C)** Perceptual boundary choice and completion: like-oriented layer 4 simple cells with opposite contrast polarities compete (not shown) before generating half-wave rectified outputs that converge onto layer 2/3 complex cells in the column above them. Long-range interactions within layer 2/3 realize a law for boundary choice and completion that is called the bipole grouping property (Grossberg, 1984a; Grossberg and Mingolla, 1985). Just like attentional signals from higher cortex, as shown in **(B)**, boundary groupings that form among bipole cells in layer 2/3 also send activation into the folded feedback path, to enhance their own positions in layer 4 beneath them via the 6 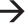 4 on-center, and to suppress input to other groupings via the 6 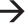 4 off-surround. There exist direct layer 2/3 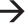 6 connections in macaque V1, as well as indirect routes via layer 5. **(D)** Top-down corticogeniculate feedback from V1 layer 6 to LGN also has an on-center off-surround anatomy, similar to the 6 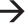 4 path, and realizes the ART Matching Rule from V1 to LGN. The on-center feedback selectively enhances LGN cells that are consistent with the activation that they cause, and the off-surround contributes to length-sensitive (endstopped) responses that facilitate grouping perpendicular to line ends. **(E)** The entire V1/V2 circuit: V2 repeats the laminar pattern of V1 circuitry, but at a larger spatial scale. In particular, the horizontal layer 2/3 connections have a longer range in V2, allowing above-threshold perceptual groupings between more widely spaced inducing stimuli to form. V1 layer 2/3 projects up to V2 layers 6 and 4, just as LGN projects to layers 6 an 4 of V1. Higher cortical areas send feedback into V2 which ultimately reaches layer 6, just as V2 feedback acts on layer 6 of V1. Feedback paths from higher cortical areas straight into V1 (not shown) can complement and enhance feedback from V2 into V1. Top-down attention can also modulate layer 2/3 pyramidal cells directly by activating both the pyramidal cells and inhibitory interneurons in that layer. The inhibition tends to balance the excitation, leading to a modulatory effect. These top-down attentional pathways tend to synapse in layer 1. Their synapses on apical dendrites in layer 1 are not shown, for simplicity. Reprinted with permission from Raizada and Grossberg (2001).

More recent versions of ART have shown how predicted ART mechanisms may be embodied by identified cells in laminar microcircuits of the cerebral cortex. Laminar cortical models for vision (Figure 2), called LAMINART models (e.g., Grossberg, 1999; Grossberg and Raizada, 2000; Raizada and Grossberg, 2003; Grossberg and Swaminathan, 2004; Cao and Grossberg, 2005, 2012; Grossberg and Yazdanbakhsh, 2005); for cognitive information processing, called the LIST PARSE model (Grossberg and Pearson, 2008); and for conscious speech processing, called the cARTWORD model (Grossberg and Kazerounian, 2011; Kazerounian and Grossberg, 2014), have all been developed using variations of the same canonical laminar circuitry. A variant of the LAMINART model, called the Synchronous Matching ART, or SMART, model (Grossberg and Versace, 2008), proposed how a thalamocortical mismatch that is mediated by the non-specific thalamus and the nucleus basalis of Meynert may increase vigilance via a non-specific burst of arousal that releases ACh to wide areas of neocortex. Before detailing how this is proposed to happen, some of the basic circuitry for realizing top-down attentional matching needs to be specified.

## 7. Attention is realized by top-down, modulatory on-center, off-surround networks

What kind of top-down attentional matching circuits support a self-stabilizing memory, and thus a solution of the stability-plasticity dilemma? Grossberg (1980) proposed that top-down on-center off-surround networks carry out the requisite matching properties. Carpenter and Grossberg (1987) went further to mathematically prove that the simplest matching circuit that can solve the stability-plasticity dilemma is a top-down, modulatory on-center, off-surround network. The modulatory on-center can sensitize, or prime, cells within the category prototype, but not fully fire them under most conditions, whereas the off-surround can inhibit cells that are not in the on-center. This kind of circuit realizes the excitatory matching that was described in Section 4.1 and Figure 1. Circuits of this type are said to obey the ART Matching Rule.

All the predicted properties of the ART Matching Rule have received behavioral, anatomical, and neurophysiological support; see Raizada and Grossberg (2003) for a review. The competitive dynamics of attention are popularly called “biased competition” (Desimone, 1998). There is also a growing consensus about the exact mathematical form that attentional circuits should take. For example, the form that was used for such attentive matching in explaining perceptual categorization data (e.g., Gove et al., 1995; Bhatt et al., 2007) was also used in the “normalization model of attention” (Reynolds and Heeger, 2009). Reynolds and Heeger (2009) expressed ART matching as an algebraic equilibrium equation. Bhatt et al. (2007) expressed it in terms of real-time neural dynamics from which its equilibrium equation was derived.

The LAMINART model predicts how the ART Matching Rule may be realized in laminar visual cortical circuits by identified neurons (Grossberg, 1999; Raizada and Grossberg, 2003). As shown in Figures 2B,E, corticocortical feedback axons from layer 6 of a higher area terminate in layer 1 of a lower cortical area, where they excite apical dendrites of layer 5 pyramidal cells whose axons send collaterals into layer 6. The feedback is then “folded” back from layer 6 to layer 4 via a modulatory on-center, off-surround network. This “folded feedback” circuit realizes the top-down, modulatory on-center, off-surround circuit of the ART Matching Rule. A variety of anatomical and neurophysiological data support the predicted properties of this circuit; see Grossberg and Raizada (2000), Grossberg and Versace (2008), and Raizada and Grossberg (2001) for descriptions and simulations of these data. In particular, habituative transmitter gates (Section 4.6) are predicted to occur at the synapses of the layer 4 on-center off-surround network, among other parts of the cortex, where their reset properties help to explain and simulate several different types of developmental, perceptual, and learning data (Francis and Grossberg, 1996; Grossberg and Seitz, 2003; Grossberg and Versace, 2008).

## 8. Mismatch, reset, and search in laminar cortical circuits

**Figure 3:**
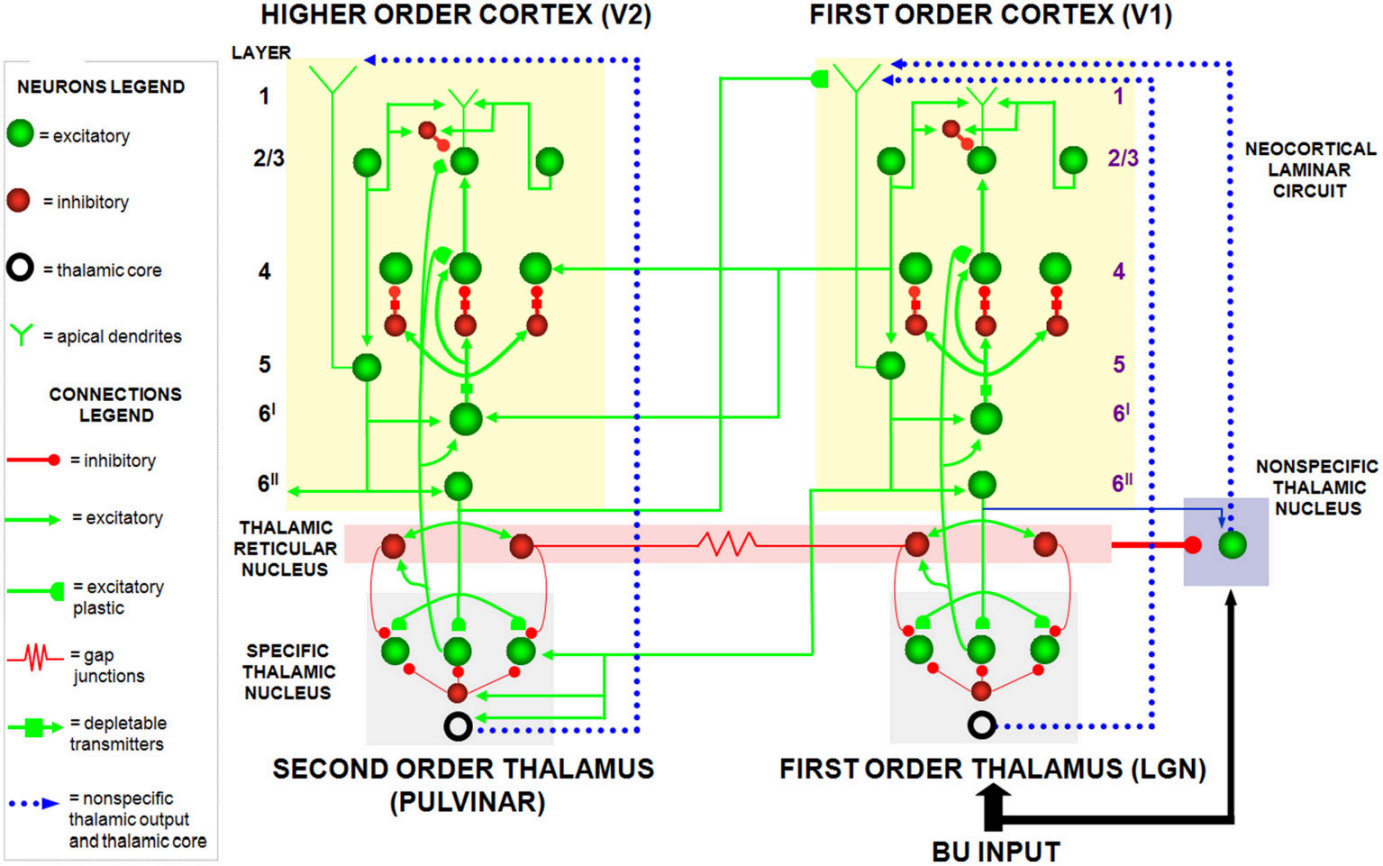
**The SMART model**. The specific thalamus (for example, the lateral geniculate nucleus, or LGN, and pulvinar) receives bottom-up (BU) input from the periphery, and top-down feedback from the cerebral cortex, where bottom-up and top-down information is matched. For example, LGN receives feedback from V1 and pulvinar from V2. Top-down feedback from the cerebral cortex also excites the thalamic reticular nucleus which provides global inhibition to the specific thalamic nucleus to suppress mismatching features in the sensory input. The non-specific thalamic nucleus receives a copy of the bottom-up sensory information, as well as inhibition from the thalamic reticular nucleus. When a mismatch occurs, the inhibition from the thalamic reticular nucleus decreases, leading to an arousal burst (dotted arrow from non-specific thalamic nucleus) that is broadly distributed across layer 1 of the cerebral cortex. This arousal burst leads to reset and search for alternative recognition codes in the cerebral cortex. Repeated mismatches achivate projections of the non-specific thalamic nucleus to the Nucleus Basalis of Meynert (see **Figure 4**), which in turn release ACh in the cerebral cortex. Modified with permission from Grossberg and Versace (2008).

The SMART model (Grossberg and Versace, 2008) further develops the LAMINART explanation of how laminar cortical circuits can reset ongoing activity to search for a better-matching visual category in response to a predictive mismatch, and additionally proposes how acetylcholine can modulate vigilance, and thus the criteria that trigger search. The SMART model (Figure 3) refines the LAMINART circuits in Figure 2 by subdividing layer 6 into the two sublamina, namely 6^I^ and 6^II^. It also models a hierarchy of cortical levels (e.g., V1 and V2) as they interact via spiking neurons with first-order (e.g., LGN) and second-order (e.g., pulvinar; Sherman and Guillery, 2001; Shipp, 2003) specific thalamic nuclei and non-specific thalamic nuclei (van der Werf et al., 2002).

In the SMART model, a bottom-up input from a layer 6^I^ cell activates a direct excitatory pathway to layer 4 and to layer 6^I^-to-4 inhibitory interneurons. The signals in these pathways are gated by activity-dependent habituative transmitters: Neurotransmitter in these pathways is released in an activity-dependent way to activate layer 4 target cells, and transmitter recovery is slow relative to its release rate. The net post-synaptic EPSP thus decreases through time to a habituated firing level after an initial activity burst (Beierlein et al., 2002). Despite the fact that larger inputs cause greater habituation, synaptic transmission remains unbiased, and stronger inputs produce bigger steady-state EPSPs, as was proved mathematically in Grossberg (1972, 1980).

As in earlier ART models, top-down corticothalamic feedback in SMART obeys the ART Matching Rule. In other words, it is realized by a top-down, modulatory on-center, off-surround circuit whose on-center determines the attentional focus that selects, enhances, and synchronizes behaviorally relevant, bottom-up sensory inputs (match), and whose off-surround suppresses inputs that are irrelevant (mismatch).

Thalamocortical dynamics repeat key properties, albeit with suitable specializations, at multiple levels of processing in SMART. In particular, the processing dynamics that occur between LGN and V1 are homologous to the dynamics between the pulvinar nucleus and V2, and beyond (Salin and Bullier, 1995; Callaway, 1998). Thus, top-down feedback from layer 6 of V2 to the pulvinar can match the bottom-up input pattern from V1 to the pulvinar in a manner similar to how top-down feedback from layer 6 of V1 to LGN matches retinal input to the LGN.

SMART refines the long-standing ART proposal (Grossberg, 1980) that the thalamic reticular nucleus (TRN) realizes the off-surround that is used during thalamic matching. The TRN forms a shell around the lateral and dorsal portions of the thalamus, that lies within the axonal path connecting the thalamus and the cortex (Guillery and Harting, 2003). TRN afferents are mainly derived from branches of bottom-up axons from the thalamus to the cortex, or branches of top-down axons from cortical layer 6 to its specific thalamic nucleus. TRN cells are GABAergic, and are reciprocally linked by both chemical inhibitory projections and electrical synapses (Landisman et al., 2002). Inhibitory top-down TRN feedback to the thalamus balances top-down cortical layer 6 excitatory signals at their shared target cells. As a result, the excitatory signals have only a modulatory effect on these cells (Guillery and Harting, 2003) when there are no other active inputs. The TRN hereby plays an important role in suppressing unmatched sensory features during visual learning and recognition.

SMART proposes how a memory search may be controlled by interactions between specific thalamic nuclei, non-specific thalamic nuclei, and the cerebral cortex, in particular how a burst of mismatch-mediated non-specific arousal may be triggered. The non-specific thalamus—notably, the midline and central lateral thalamic nuclei—is sensitive to the degree of mismatch between cortical expectations and sensory stimuli (Kraus et al., 1994). A big enough mismatch at a specific thalamic nucleus can generate a novelty-sensitive activity burst at a non-specific thalamic nucleus (van der Werf et al., 2002) that is broadcast non-specifically to the superficial layers of the cerebral cortex, notably layer 1. This non-specific signal propagates from layer 1 dendrites to their layer 5 cells, then to layer 6, and finally to layer 4 via habituatively-gated signals. The activity-dependence of habituation in different pathways enables the non-specific arousal burst to cause selective reset of active layer 4 cells (Section 4.6).

Grossberg and Versace (2008) did model simulations showing that the human mismatch negativity (MMN) event-related potential has features that are consistent with these mismatch-mediated events. Indeed, MMN properties are related to an earlier ART prediction that the mismatch, arousal, and reset events that occur during an ART search (Figure 1) correspond to different human scalp-recorded Event Related Potentials, or ERPs, and that these ERPs should co-occur, as they do in the ART search cycle, if they occur at all. In particular, Processing Negativity, N200 (a component of which is MMN), and P300 ERPs were predicted to correspond to match, arousal, and STM reset events at various levels of thalamocortical processing (Grossberg, 1978, 1980, 1984c). This prediction was tested and supported by ERP experiments (Banquet and Grossberg, 1987). In these experiments, an oddball paradigm used low and high tones within a choice reaction time task. As predicted, components of the P120, N200, and P300 ERPs co-occurred and behaved like mismatch, arousal, and STM reset events.

## 9. Acetylcholine modulates vigilance, learning, and generalization via the nucleus basalis

SMART also further specified how vigilance control, and thus the learning of concrete vs. or abstract recognition categories, is realized in laminar cortical circuits. As noted above, an arousal burst can sometimes activate layer 5, leading to reset of layer 4 and search for a new recognition category. If the sensitivity of layer 5 to such an arousal burst can be modulated by predictive success, then a process like match tracking can be realized.

**Figure 4:**
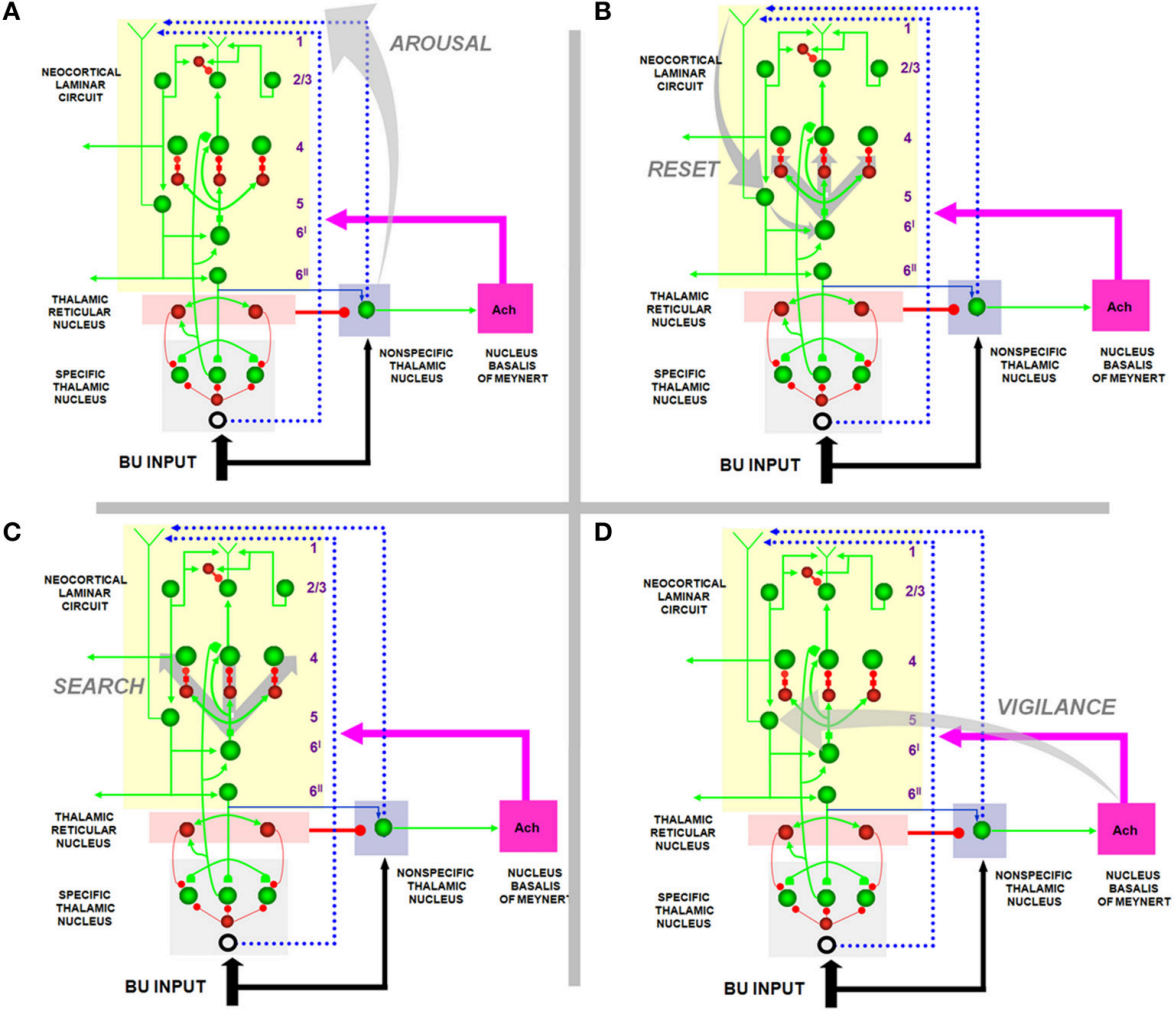
**How the SMART model refines ART and LAMINART search mechanisms. (A)** Arousal: In response to a mismatch, the non-specific thalamic nuclei activation non-specific projections to apical dendrites in layer 1 of layer 5 cells; **(B)** reset: habituative synapses in the layer 6^*I*^ → 4 pathways respond to arousal increases in the layer 5 → 6^*I*^ → 4 pathways with reset of previously active cells; **(C)** search: reset enables new cells to get activated that, possibly after several reset cycles, can better represent the current inputs; **(D)** vigilance control: ACh release occurs in the cortex due to mismatch-activated signals from the nucleus basalis of Meynert. High levels of ACh can increase the excitability of layer 5 pyramidal neurons by reducing afterhyperpolarization currents and spike adaptation, thereby increasing vigilance and facilitating reset by requiring a higher degree of match between bottom-up and top-down representations to keep the arousal signal small. Reprinted with permission from Grossberg and Versace (2008).

SMART provides testable predictions about how this happens (Figure 4). These predictions already have considerable experimental support, albeit support that is not generally described in terms of vigilance control and the generality of category prototypes: The non-specific thalamic nucleus can activate the nucleus basalis of Meynert (van der Werf et al., 2002), which is an important main source of cholinergic input to the cerebral cortex. Both *in vitro* data (Saar et al., 2001) and computer simulations of isolated model layer 5 pyramidal cells show how ACh can regulate after-hyperpolarization (AHP) currents and, with them, the excitability of layer 5 cortical cells. Indeed, a steady depolarization current causes rat pyramidal cell firing to rapidly habituate. In opposition to this, injection of the ACh agonist carbachol reduces the adaptation (Saar et al., 2001). ACh can hereby modulate, through the reduction of AHP and the prevention of spike adaptation, the excitability of layer 5 pyramidal neurons. In so doing, ACh can regulate the amount of thalamic mismatch that can be tolerated by the cortical area before excitability increases. Vigilance may be increased by high levels of ACh through its effect of reducing spiking adaptation and thereby facilitating reset. By imposing a more demanding criterion of match between bottom-up and top-down representations before resonance and learning can occur, higher levels of ACh force learning of more concrete categories than would occur without it. For this to work, ACh concentration transients must act on the timescale of behavioral episodes, as they have been report to do (Parikh et al., 2007; Sarter et al., 2009). They must also vary in a task-dependent manner that correlates with attentional demands. This property has been confirmed by microdialysis (Marrosu et al., 1995; Arnold et al., 2002) and newer techniques (Parikh et al., 2007). A role for ACh in vigilance control is also consistent with the fact that the cholinergic blocker scoplamine reduces novelty discrimination in rats (Ballaz, 2009), and that lesions in rats of the nucleus basalis of Meynert have little impact on learning rate, except when a high degree of featural overlap occurs between the categories to be learned (Botly and De Rosa, 2007, 2009), and thus higher vigilance is required. Also consistent is the fact that the cholinergic blocker scopolamine diminishes learning of overlapping word pairs more than non-overlapping pairs (Atri et al., 2004).

Recent modeling work demonstrates how acetylcholine can control the shape of neural input/output transfer functions by regulating AHP currents, defined as spike-dependent, hyperpolarizing currents that occur following action potentials (Palma et al., 2012a,b). Three main classes of AHP currents have been identified in a variety of mammalian species and brain regions: fast (fAHP), medium (mAHP), and slow (sAHP, Storm, 1987; Schwindt et al., 1988; Lorenzon and Foehring, 1992; Lee et al., 2005). Simulations in multi-compartment, spiking cortical cells show that ACh can shift the neuron's transfer function by diminishing sAHP and mAHP, while boosting fAHP (Palma et al., 2012a,b), as supported by physiological recordings directly (Storm, 1987; Lorenzon and Foehring, 1992; Vogalis et al., 2003) or indirectly (Prakriya et al., 1996; Bordey et al., 2000; Matthews et al., 2009). The net effect of ACh stimulation is a leftward shift of the transfer function of neurons. This lowers the range of competition and temporally expands the number of competitive candidates in a target neural population, as was earlier demonstrated in rate-based models (Grossberg, 1973; Ellias and Grossberg, 1975). It also accelerates the rate of competition. These effects could promote pattern differentiation, as observed in the primary auditory cortex of the rat (Pandya et al., 2005). Spiking network models (Palma et al., 2012b) confirm that the net result of an increase of ACh release is a “choice,” or code sharpening, in the target network. This mechanism provides the modulatory control necessary to ensure that the sharpness of the neural code that is learned in a cognitive or motor area supports the behavioral success of the organism.

## 10. Learning a multimodal movement map

### 10.1. Merging visual, auditory, and planned movement commands by learning

**Figure 5:**
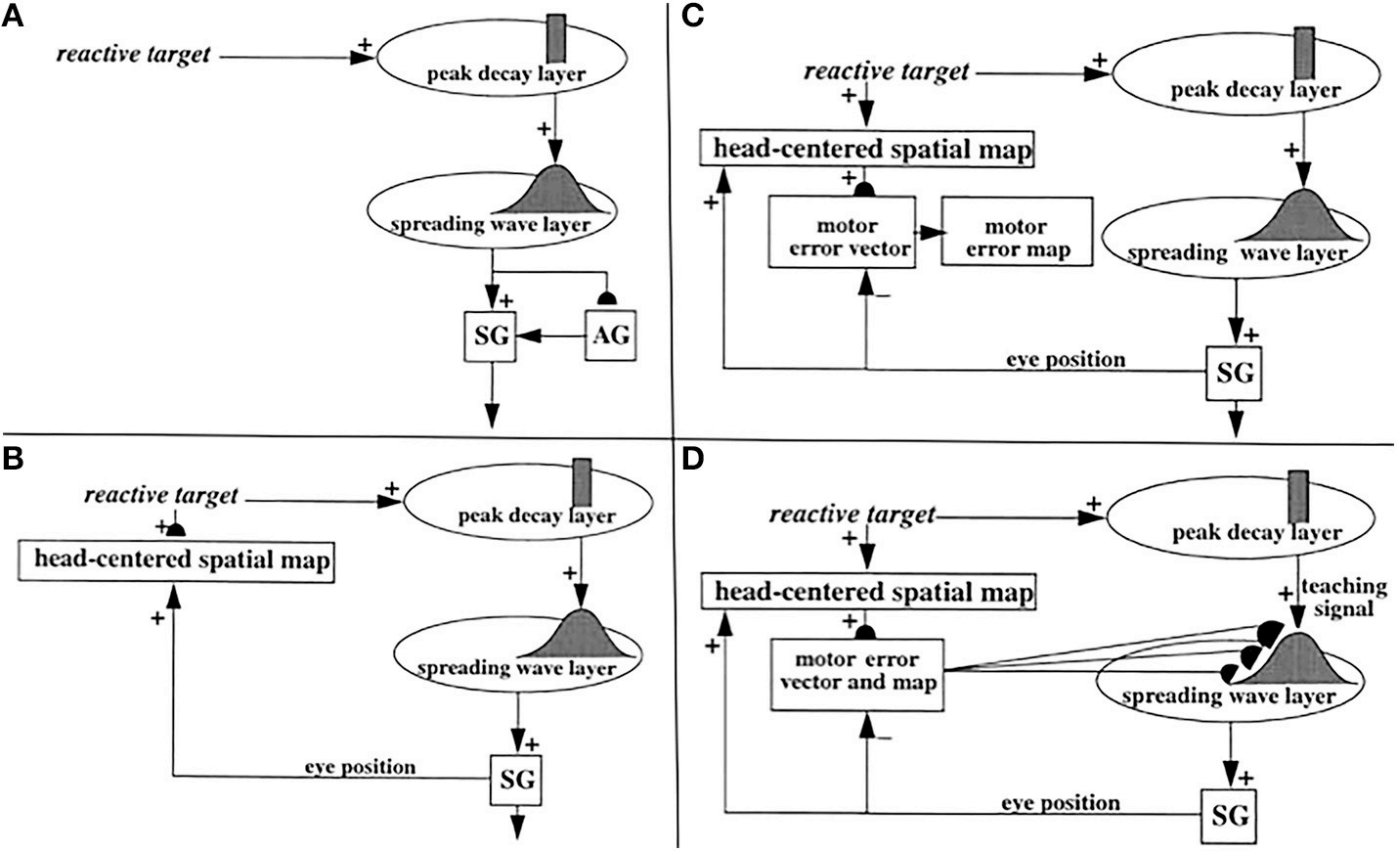
**(A)** Initially, saccades are executed reactively to targets that are registered on the retina. These retinotopic signals map topographically into a motor error map (Grossberg and Kuperstein, 1986, Chapter 3). Motor error signals activate map locations in the peak decay (PD) layer of burst cells that, in turn, topographically excite the spreading wave (SW) layer of buildup cells. The term “spreading wave” designates the spreading activity that occurs at buildup cells during an eye movement. The adaptive gain (AG) properties of the cerebellum enable accurate reactive saccades to be made via the saccade generator (SG) in the peripontine reticular formation. **(B)** A corollary discharge from tonic cells of the SG provide an accurate measure of current eye position. The eye position signal combines with a target position signal from the retina, coded in retinotopic coordinates, to generate a head-centered representation of the target position. Such a head-centered spatial map can be used as a source of auditory, intentional and memory-based movement commands, since these signals are also coded in head-centered coordinates. **(C)** Target positions in head-centered coordinates are adaptively mapped to a gaze motor error in retinotopic coordinates in order to map onto the SC motor error map in a dimensionally consistent way. This transformation takes place in the model in three steps. First, the transformation between a head-centered target position and a motor error vector (viz., the direction and amplitude of the desired eye movement) is learned. This transformation is learned by computing the difference between the head-centered target position and the final eye position after a reactive movement terminates. This computed difference is a motor error vector. Because reactive movements are rendered accurate by cerebellar learning, the final eye position is the same as the target position after such a movement. In other words, the motor error vector between the stored head-centered target position and the final eye position should equal zero. Learning of the transformation is thus accomplished by a process that reduces the error vector to zero (Grossberg and Kuperstein, 1986, Chapter 4). This is accomplished by using the error vector as a teaching signal that alters the adaptive weights in the pathway from the cells that compute the head-centered spatial map to those that compute the motor error. Weight learning continues until the error equals zero. After learning is complete, the head-centered target position can be transformed into the corresponding motor coordinates at the motor error vector cells. The second step converts these motor vectors into locations on a topographic map, which is called the motor error map. This step transforms large activity levels in the motor vector code to caudal positions in the topographic map and small activity levels to rostral positions (Grossberg and Kuperstein, 1986, Section 6.3). **(D)** The third step is a learned transformation from the maps of the auditory, visually attentive, and planned motor errors to the map of visually reactive motor errors at the buildup cell or SW layer of the SC. Reprinted with permission from Grossberg et al. (1997).

**Figure 6:**
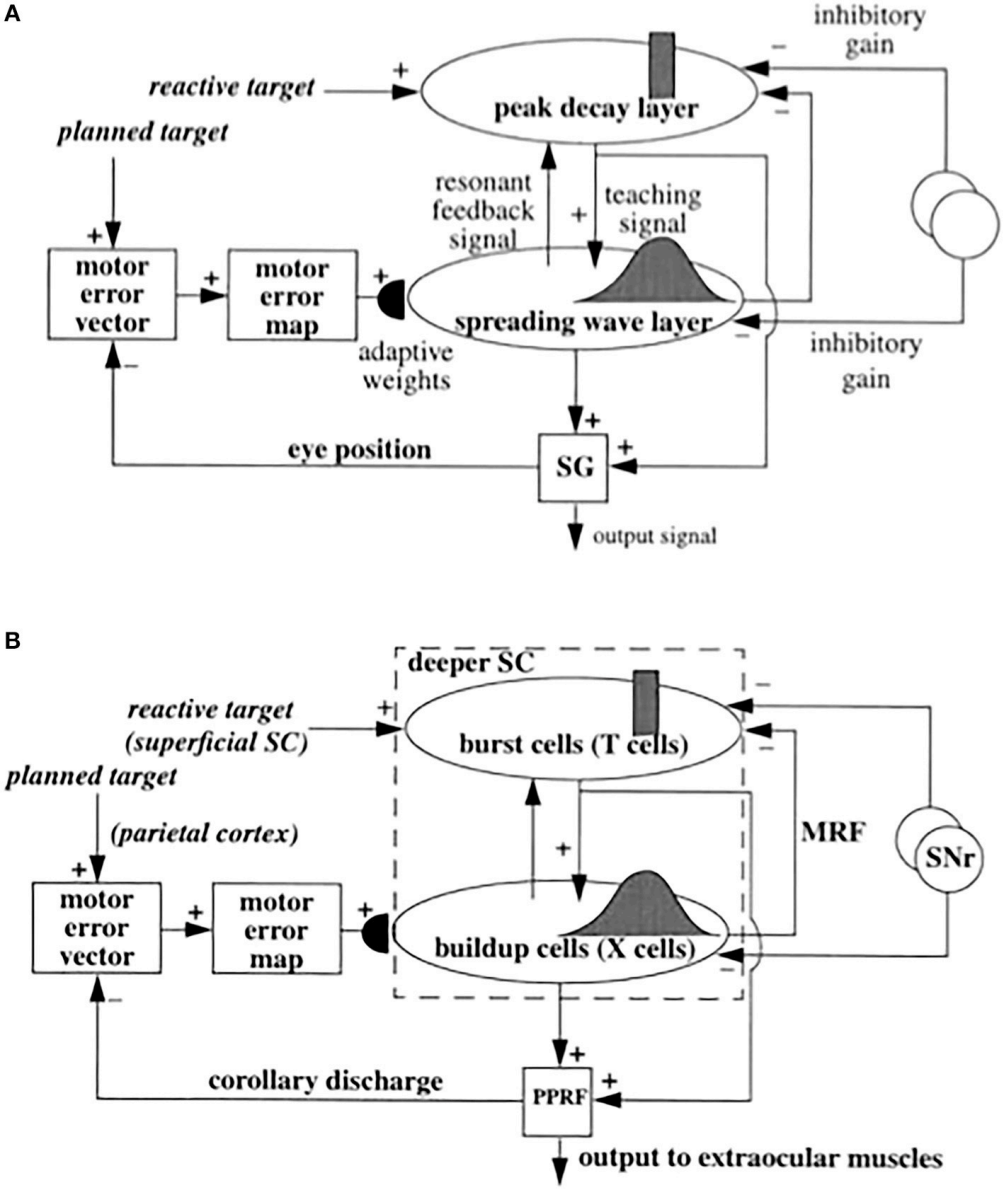
**(A)** Functional names of SACCART model connections and processes. **(B)** Anatomical and neurophysiological interpretation of SACCART model processes in **(A)**. SC, superior colliculus; superficial SC, superficial layers of the SC; deeper SC, deeper layers of the SC; SNr, substantia nigra pars reticulate; PPRF, paramedian pontine reticular formation; MRG, mesencephalic reticular formation. Reptinted with permission from Grossberg et al. (1997).

**Figure 7:**
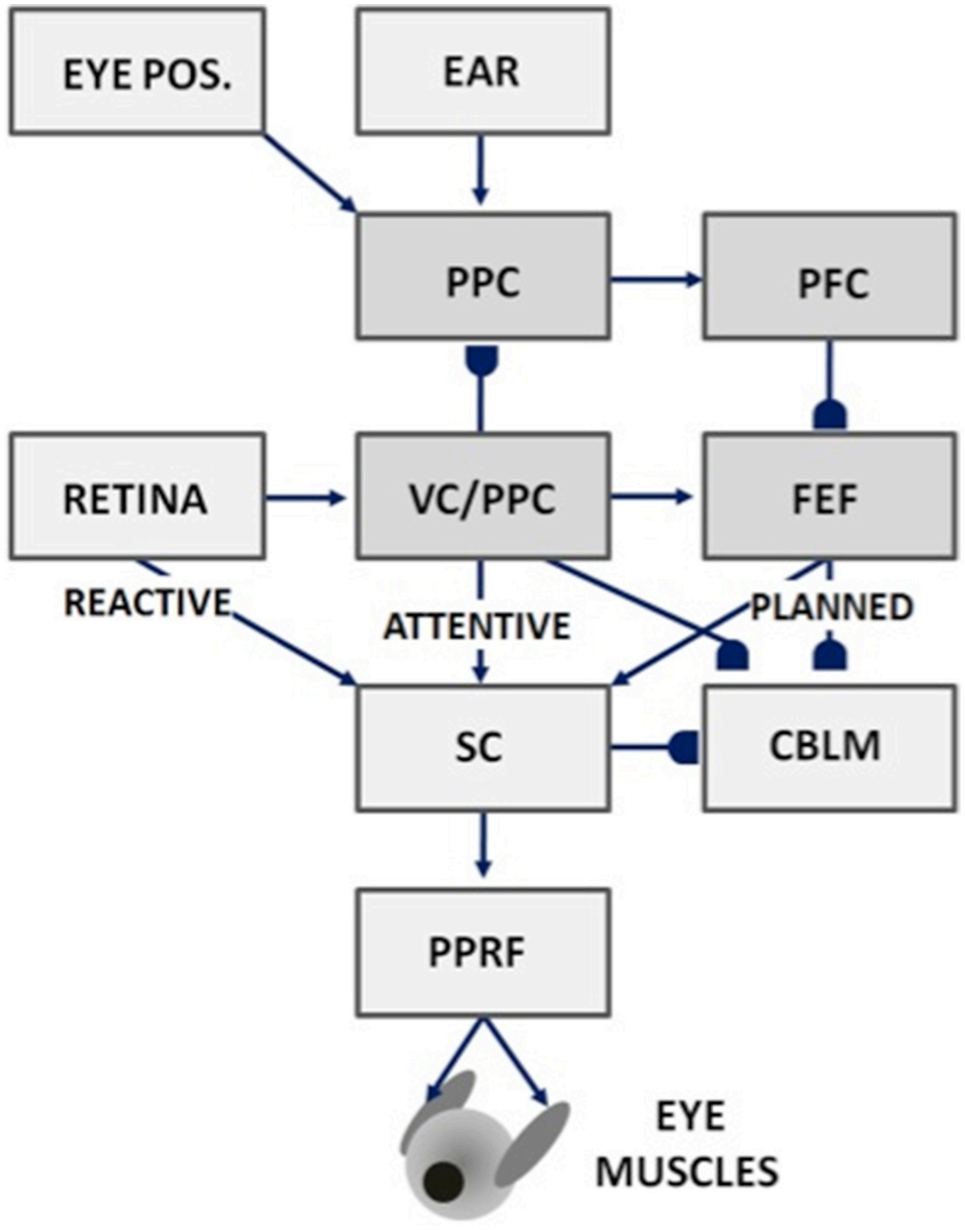
**Saccades can be made reactively to visual cues, attentively to visual or auditory cues, or planned in response to memory cues using attentive visual, parietal, and prefrontal cortical signals, as well as in response to superior colliculus, cerebellum, and reticular formation output**. Gancarz and Grossberg (1999) model how these three processing streams (reactive, attentive, and planned) learn to control accurate saccadic eye movements, despite having different maps and parameters. VC, visual cortex; PPC, posterior parietal cortex; PFC, prefrontal cortex; FEF, frontal eye fields; SC, superior colliculus; PPRF, paramedian pontine reticular formation; CBLM, cerebellum. Modified with permission from Gancarz and Grossberg (1999).

How does the brain implement attentive category learning and vigilance control in sensory-motor circuits? The SACCART model (Figures 5, 6; Grossberg et al., 1997) proposes how multiple sources of saccadic eye movement signals learn to interact to select a single position to which a saccadic eye movement will be directed. There are at least four types of saccadic movement signals: visually reactive, visually attentive, auditory, and planned (Figure 7; Gancarz and Grossberg, 1999). Visually reactive saccades are reflexive movements generated by areas of rapid visual change. Visually attentive saccades are activated by signals from an attentively-modulated region of the parietal cortex, as modeled in Fazl et al. (2009) during the learning of invariant object categories. Auditory saccades direct the eyes toward acoustic stimuli, and may be processed by the inferior colliculus and parietal cortex, among other brain regions. Planned saccades involve storage of a saccadic command in a prefrontal cortical short-term working memory, even after the cue that signals future performance of the saccade itself terminates. Read-out of such stored commands can activate saccades at a later time to intended targets; they “direct the eye at objects selected beforehand from the visual environment” (Becker, 1989).

Visually attentive, auditory, and planned representations are all computed in head-centered coordinates. A parietal head-centered map (Stricanne et al., 1996) allows visually attentive cues to cooperate or compete for attention with auditory cues. Head-centered representations do not change when eye movements occur in the absence of head or body movements. For this reason, head-centered target representations are also useful for storing several sequential target positions in short-term working memory in the prefrontal cortex and frontal eye fields, whose working memory capabilities can be used for saccadic planning (Zingale and Kowler, 1987; Goldman-Rakic, 1990, 1995; Wilson et al., 1993; Fuster, 1996).

Gancarz and Grossberg (1999) model how head-centered representations of visually attended and planned eye positions can be formed and calibrated through learning. Their model, and the subsequent refinements in articles such as Chang et al. (2014), Fazl et al. (2009), and Silver et al. (2011), also clarifies how competition within head-centered representations can choose a single target position of each type to send to the superior colliculus.

Signals for visually reactive, visually attentive, auditory, and planned saccades converge in the deeper layers of the superior colliculus, or SC (Figures 5–7), where they compete for attention in a shared multimodal target position map (Schlag-Rey et al., 1992; Stein and Meredith, 1993). To accomplish this multimodal merging of signals, the brain solves a challenging problem, since visual cues are registered in retinotopic coordinates, whereas visually attentive, auditory, and planned cues are registered in head-centered coordinates. How are these distinct coordinate systems transformed so that a particular SC map location can represent a given target position, whether it be commanded by vision, audition, or a cognitive plan? The transformation that aligns these several different types of input sources must be learned, since the parameters that characterize an individual's visual, auditory, and planning systems may change with experience throughout life. Through this map learning process, unimodal inputs to SC are aligned within the deeper layers of SC so that competitive selection, attentional focusing, decision making, and action can occur (Kowler et al., 1995; Deubel and Schneider, 1996; Grossberg et al., 1997).

The SACCART model explains how this map learning process may work (Figure 6), and hereby provides a natural functional explanation for both the peak decay and wave-like activity patterns exhibited by the burst and buildup cells, respectively, that are found in the superficial and deeper layers of the SC (Moschovakis et al., 1988; Munoz et al., 1991; Waitzman et al., 1991; Guitton, 1992; Munoz and Wurtz, 1995a,b). SACCART also explains why buildup, but not burst, cells show activation well in advance of planned saccades.

### 10.2. Calibrating visually reactive movements with visual error signals

How do saccadic eye movements from multiple types of signals learn to become accurate? Early in development, visual cues trigger saccades via a visually reactive saccadic system. These reactive eye movements are made by topographically transforming retinotopic visual signals into a motor error map (Grossberg and Kuperstein, 1986, Chapter 3). In other words, a reactive movement target signal is processed by the retina, which in turn maps it topographically into a localized activation on a motor error map in the superior colliculus (Figure 5A). This coordinate change converts positions activated on the retina into motor commands for contracting each eye's opponent muscles in approximately the direction and distance that will move the eye to that position.

The motor error signals accomplish this by activating map locations in the peak decay (PD) layer (Figure 5A) of burst cells (Figure 6) in the superficial SC layers. Burst cells then topographically excite the spreading wave (SW) layer of buildup cells in the deeper SC layers (Figures 5, 6). The term “spreading wave” is used to describe the spread of activity across the SC map that occurs continuously at buildup cells during a saccade. These reactive target coordinates at PD and SW cells are consistent with the motor error coordinates that are coded in collicular maps (Davson, 1990). Their outputs move the eyes.

These reactive movements are not necessarily accurate at first. Accuracy is achieved by compensatory signals that are computed via a side path through the cerebellum, namely the Adaptive Gain, or AG, stage in Figure 5A, which adds a learned gain to the reactive movement signal that is learned in response to a visual error signal. If a saccade is not accurate, it does not foveate the eye. Its non-foveal landing position generates visually-activated error-based teaching signals that alter cerebellar gains until the eye can make accurate visually reactive saccades, at which time the error signal equals zero (Ito, 1984; Grossberg and Kuperstein, 1986, Chapter 3; Goldberg et al., 1991; Fiala et al., 1996).

### 10.3. Auditory and planned movements base their accuracy on visually reactive learning

As modeled in the SACCART model (Grossberg et al., 1997; Figures 5, 6), the accuracy of visually attentive, auditory, and planned saccades through the SC builds on accurate visually reactive movement commands (e.g., Knudsen, 2002) by all activating the same SC map position to command a saccade to a given position, and thereby all benefiting from the learned cerebellar gain that is activated from each SC map position. For this to occur, a transformation needs to be learned from the head-centered coordinates in which auditory and visually attentive commands occur from the parietal cortex, and planned commands occur from the frontal eye fields, into the motor error coordinates into which visual signals are transformed (Jay and Sparks, 1984, 1987a,b, 1990; Schlag-Rey et al., 1992). Then targets in retinotopic and head-centered coordinates are dimensionally consistent and can compete for attention (Kowler et al., 1995; Deubel and Schneider, 1996) to choose a movement target location in motor error coordinates. That such a transformation is learned by the brain is consistent with data wherein the latency of auditory saccades depends on retinotopic motor error, as is also the case for latency to a visual target presentation (Zambarbieri et al., 1995). Gilmore and Johnson (1997) have proposed that this transformation is complete by 6 months of age in human infants.

How is this multi-modal transformation learned? When auditory, visually attentive, or planned movement vectors represent the same position as a visual target, then the former vectors learn how to map onto the SC map locations that represent the same visually reactive movement command. This map learning process occurs within the spreading wave layer (Figure 5D). At SC map positions where the signals from these auditory or planned targets disagree with the visually reactive target, then learning between these different representations is suppressed by competition by the recurrent on-center off-surround interactions across the SC. Competition across map locations helps to stabilize the map learning process by suppressing all but the winning cells. The learned map is thus not eroded by interference from multiple possible target positions and their corresponding teaching signals.

Map learning in the SACCART model may be understood in greater detail as follows: Each active burst cell outputs a topographic teaching signal to the buildup cell layer (Figures 5, 6). This teaching signal is a Gaussian distributed input centered at the position of maximal activation of the burst cell. Maximal learning occurs at the position of the Gaussian peak, whereas less learning occurs along the Gaussian flanks. Each error vector is hereby associated with a *population* of SC cells. The most active cell occurs at the map location that codes the correct saccadic direction and amplitude. New target locations that have not been practiced during development can also generate accurate saccades by using the Gaussian distribution of learning to interpolate locations that have been practiced (Sparks and Nelson, 1987; Sparks and Mays, 1990). Two other consequences that are observed in data are that saccadic averaging can occur when Gaussians that are activated by two target locations overlap (Schiller and Sandell, 1983), and buildup activity is distributed across a broad expanse of SC cells.

In order for head-centered auditory or planned movement commands to learn from this teaching signal, they need first to be converted through learning into coordinates that are compatible with the motor error coordinates within the SC deeper layers. The next section proposes how this may be done. Assuming that it has already been done, map learning takes place when a visual cue onset is coded by both the head-centered and visually reactive pathways (Figure 5B). Consistent simultaneous activity in both pathways allows the head-centered representation (after being transformed into coordinates that are compatible with motor error, as in Figure 5C) to activate adaptive connections that sample the Gaussian teaching signal (Figure 5D) on a number of learning trials, as statistically uncorrelated locations get suppressed by competition across the layers. An auditory or planned target may hereby be adaptively transformed from a head-centered representation to the corresponding gaze motor error in the visually reactive motor error map. The motor error map in this model layer has properties that resemble the directional maps that are found in deep layers of the SC (Sparks and Mays, 1980).

**Figure 8:**
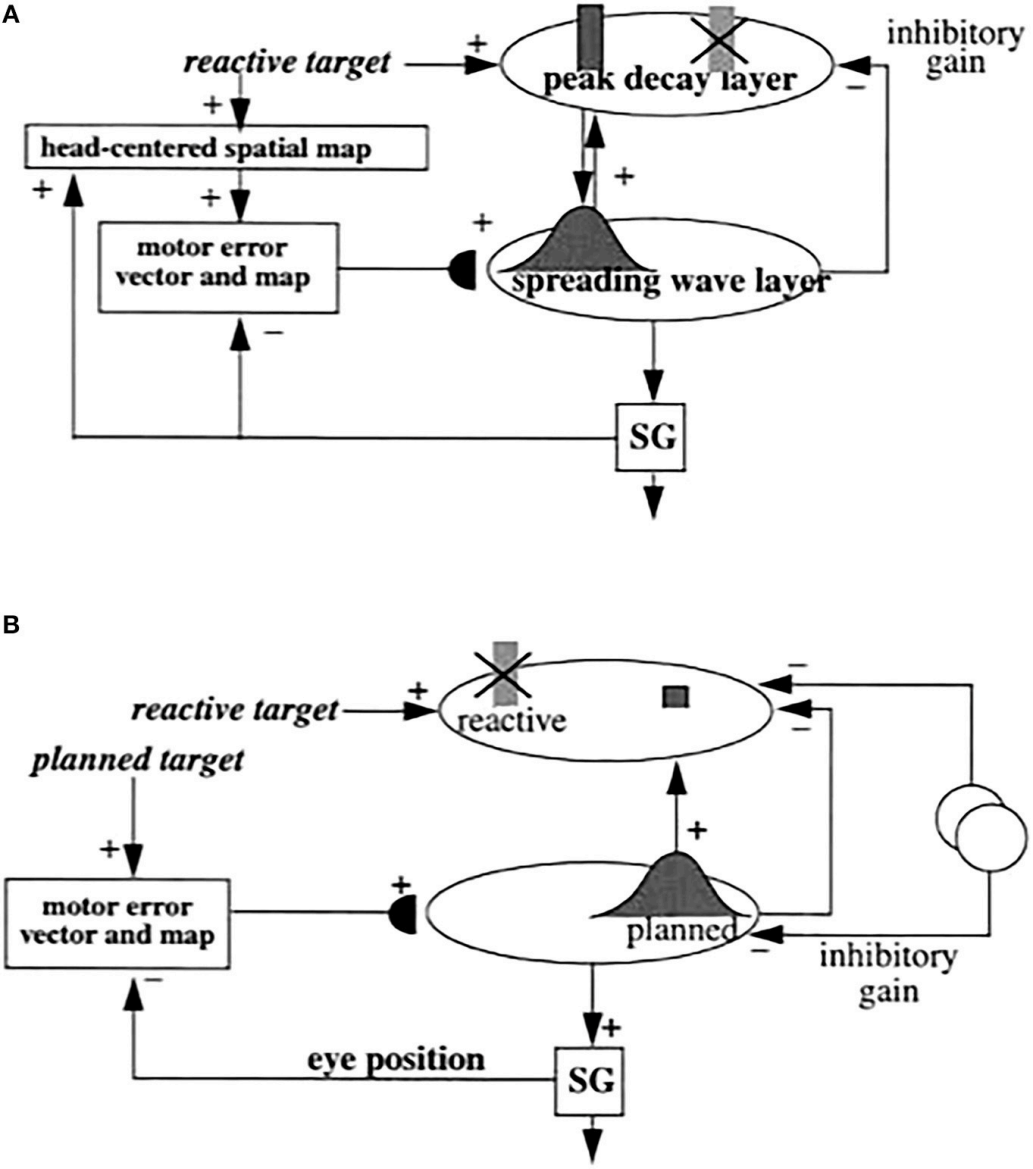
**(A)** When the peak decay and spreading wave layers represent the same target position, they can resonate via a positive feedback loop. In addition, the spreading wave layer inhibits activities that represent other target positions within the peak decay layer. The total recurrent shunting on-center off-surround network embodies the ART Matching Rule, and can rapidly choose a preferred target position while inhibiting less activated ones. **(B)** How a planned target position command can inhibit a reactive movement command that represents a different target position by using the off-surround of the ART Matching Rule. Reprinted with permission from Grossberg et al. (1997).

After learning occurs, when these various input sources on later occasions do not represent the same target positions, they compete across the SC map (Kowler et al., 1995; Deubel and Schneider, 1996) to select winning cells whose activity generates a focus of attention and an output that drives a saccadic eye movement to the winning position (Figure 8). In particular, Figure 8A illustrates how a resonance that is supported by reciprocal connections between the peak decay and spreading wave layers (cf., Ghitani et al., 2014) can choose a winning motor map position; drive learning of the multimodal map adaptive synapses from auditory, planned, and visually-attentive motor error maps to the SC motor error map; and inhibit other SC map positions via the ART Matching Rule off-surround. In particular, Figure 8B illustrates how, when the positions encoded by a reactive and planned eye movement command are not the same, whether during map learning or the unfolding of a saccadic movement, the buildup-to-burst inhibitory feedback can erode the mismatched activity at the peak decay layer.

### 10.4. Transforming a head-centered representation into a motor error vector through learning

**Figure 9:**
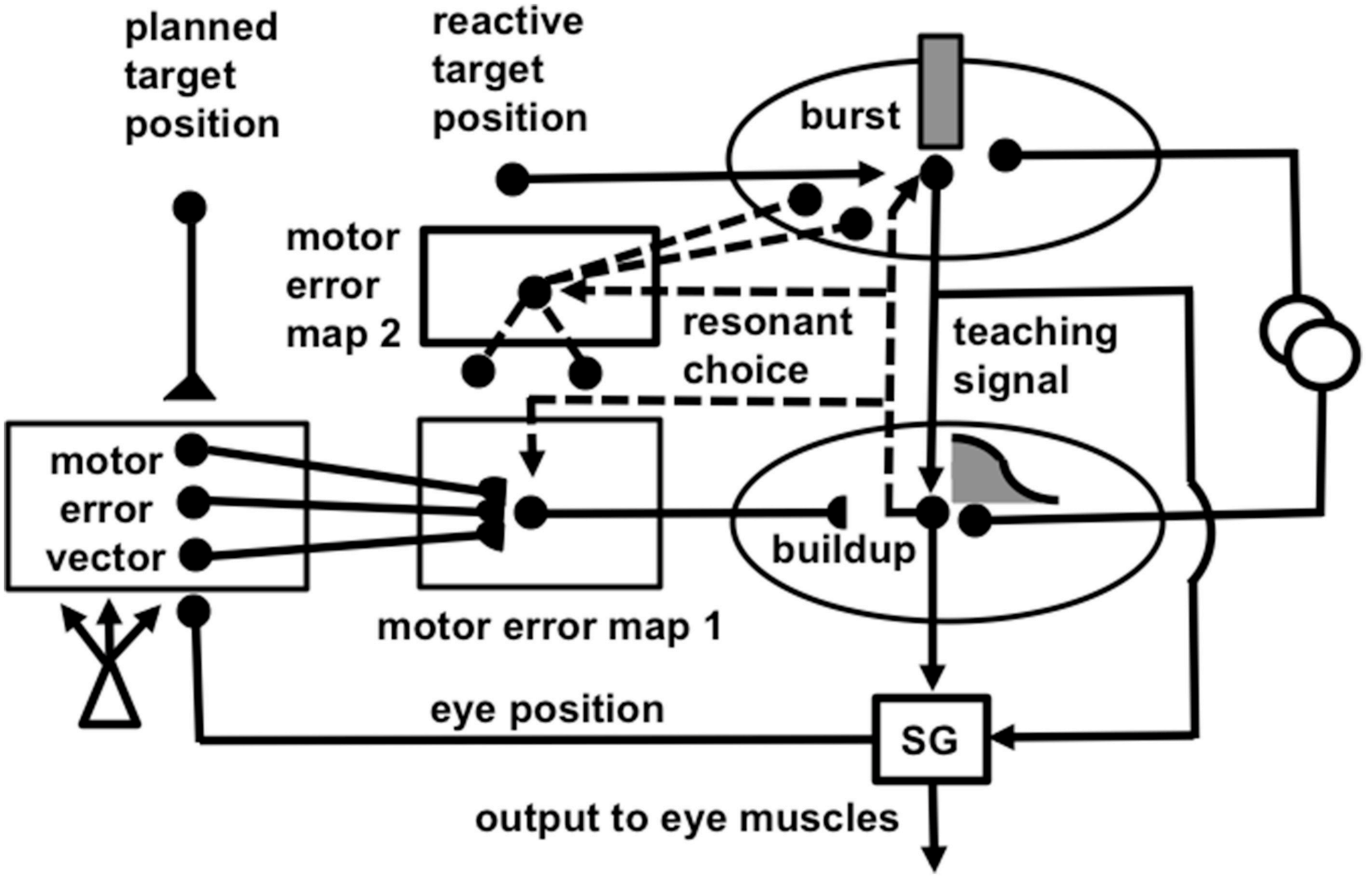
**A variant of Figures 5, 8 in which the recurrent on-center and off-surround of the ART Matching Rule are embodied in different brain regions, called motor error map 1 and motor error map 2, respectively**. Symbols: semi-disk synapses, adaptive excitatory synapses that learn vector-to-map and map-to-map associations; reverse triangle synapses, inhibitory synapses that calibrate map-to-vector motor gains using the motor error vector as a teaching signal; triangular synapses, excitatory non-adaptive synapses; circular synapses, inhibitory non-adaptive synapses; dashed lines, recurrent pathways that support resonant matching and choice; open circles, inputs from the substantia nigra pars reticulata that open movement gates; open triangle, postural gate (e.g., signal from a pauser cell) that enables motor error vector learning when the system is at a fixed posture.

How are auditory, visually attentive, and planned signals, that are coded in head-centered coordinates, transformed through learning into coordinates that can be consistently mapped into the visually-activated position of the same object in the SC motor error map? The SACCART model predicted and simulated how this may be accomplished (Figures 5, 6). The current article refines the description of the anatomical connections whereby *multiple* simultaneously active head-centered representations carry out such a learned transformation without degrading map learning (Figure 9). This anatomical description preserves all the functional properties of the previous analysis, but also is consistent with data about the parabigeminal nucleus in mammals (Graybiel, 1978; Baleydier and Magnin, 1979; Sherk, 1979; Watanabe and Kawana, 1979; Cadusseau and Roger, 1985; Mufson et al., 1986) and the isthmic nuclei in birds (Major et al., 2000; Wang, 2003; Wang et al., 2004; Maczko et al., 2006; Marín et al., 2007; Asadollahi et al., 2010), and suggests testable predictions about the functional roles played by the anatomy and neurobiology of these structures. The main anatomical refinement (Figure 9) is to segregate the excitatory and inhibitory feedback signals between the multimodal maps and the SC motor error map into two separate motor error map regions (labeled motor error map 1 and motor error map 2 in Figure 9), respectively. Finally, it enables a more refined discussion to be given of the possible role of ACh modulation in supporting saccadic choice and reset.

**Figure 10:**
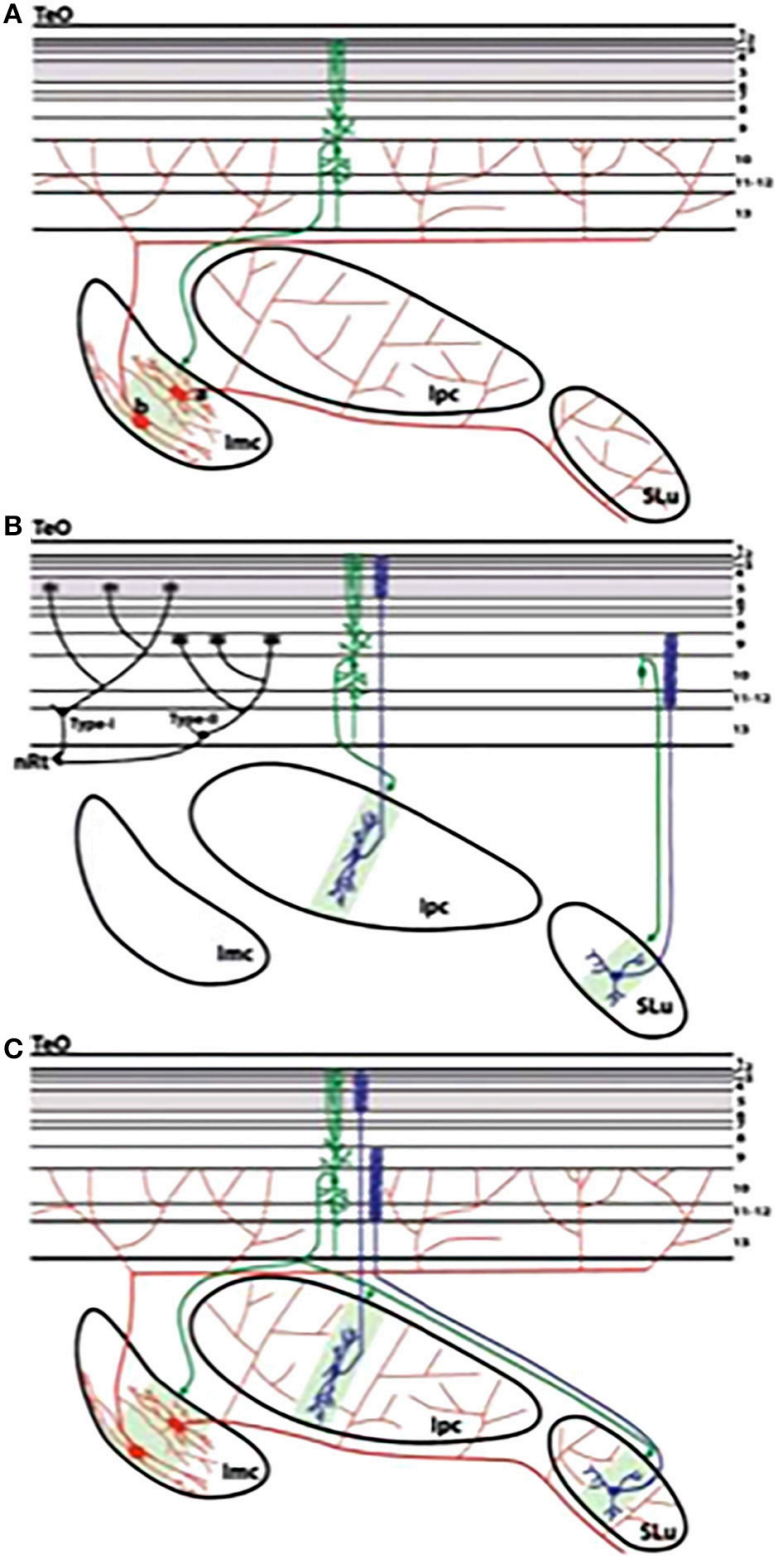
**Summary of the neuronal circuitry of the TeO (OT), Imc, Ipc, and SLu. (A)** Imc receives a coarse topographical input from the radial tectal neurons in layers 10–11. Imc-Is neurons **(A)** and Imc-Te neurons **(B)** project widely upon Ipc/SLu and TeO, respectively. **(B)** Ipc and SLu are retinotopically and reciprocally connected with the TeO. The paintbrush endings of Ipc and SLu neurons terminate within retinorecipient and non-retinorecipient tectal layers, respectively, ending in proximity to type I and type II stratu griseum central (SGC) neurons (Luksch et al., 1998; Major et al., 2000; Marín et al., 2003). **(C)** Summary of the intercircuitry among the TeO, Imc, Ipc, and SLu. The gray shadings of the TeO indicate retinorecipient tectal layers. The green shadings within Imc, Ipc, and SLu indicate the tectal terminal projection. The Ipc neuron is based on the reconstruction of an intracellular filled neuron (Y. Wang, unpublished observations). The SLu neuron is reproduced from the Güntürkün (1987) Golgi study. The motor error map 1 in Figure 9 plays the role of Ipc and the motor error map 2 plays the role of Imc. TeO, optic tectum, also OT; Ipc, nucleus isthmi pars parvocellularis; Imc, nucleus isthmi pars magnocellularis; SLu, nucleus isthmi pars semilunaris; nRt, nucleus rotundus. Reprinted with permission from Wang et al. (2004).

This anatomical refinement uses a variant of the ART Matching Rule that is supported by anatomical data (e.g., Wang et al., 2004). In this variant, the modulatory property of the on-center feedback is achieved by having no positive feedback at all, thereby realizing the modulatory balance between excitation and inhibition in the on-center feedback signal in the simplest possible way. The off-surround provides negative feedback to all other map positions than the on-center of the chosen target position (Figure 10). In other words, it defines an “anti-topographic projection” (Gutiérrez-Ibáñez et al., 2014). See Section 10.7 for further discussion.

This simplest variant of the ART Matching rule has been used to successfully model other parts of the brain. For example, the 3D FORMOTION model proposes how the brain computes a globally consistent representation of object motion direction and speed out of the many locally inconsistent directional and speed signals that are initially computed due to computational limitations that are caused by the aperture problem (Berzhanskaya et al., 2007). This simplest ART Matching Rule enables the correct object motion direction *and* speed to be co-selected by a resonant ART Matching Rule feedback loop that occurs between model cortical areas MT and MST. Here, too, there is a type of multimodal map learning. In the case of visual motion perception, this multimodal learning occurs within V2-to-MT connections between representations of 3D visual form in cortical area V2 and representations of 3D visual motion in MT. Such a *formotion* interaction enables representations of 3D form that are computed in the What cortical processing stream, including cortical areas V1 and V2, to be tracked as they move through time by motion mechanisms in the Where cortical processing stream, including cortical areas MT and MST.

In order to learn the multimodal SC map, first a transformation is learned between a head-centered target position and the corresponding *motor error vector*, which represents the direction and amplitude of the desired eye movement needed to foveate the target. Such vectors are an important computational strategy for downloading movement commands by the cerebral cortex; e.g., Georgopoulos (1994). This transformation is learned by computing the *difference* between an attended target position in a head-centered spatial map and a motor representation of the final eye position after an accurate visually reactive eye movement foveates that target (Figure 5C). To understand how this happens, suppose for definiteness that the attended input is auditory. Then the inhibitory “corollary discharge,” or efference copy, signals representing the final eye position are subtracted from adaptive excitatory signals from the auditory head-centered map source at the motor error vector processing stage (Figure 5C). This computed difference is a motor error vector. It is a *motor* error vector because it is computed in the same motor coordinates as the commands to move the opponent eye muscles within the saccade generator (SG) in the peripontine reticular formation (PPRF). These motor coordinates are imposed by subtracting the efference copy signals of the eye movement commands at the stage that computes the motor error vector. The movement commands are *head-centered* because the eyes move within the head. The target position in the head-centered spatial map is transformed into a *vector* of oculomotor coordinates that represent the current eye position by the learning process that is described in the next paragraph. By this learned transformation, the head-centered auditory, visually attentive, or planned input vectors, as well as their efference copies, are computed in head-centered *motor* coordinates.

Because visually reactive movements are rendered accurate by cerebellar learning, the head-centered and efference copy motor vectors both represent the same position in space after the movement to the external target occurs. If both sets of signals are calibrated correctly as a result of learning, the motor error vector should equal zero after the efference copy motor vector is subtracted from the learned motor vector that is read-out from the head-centered target position. If it is not zero, then the motor error vector serves as a mismatch teaching signal that changes the adaptive weights in the pathway from the head-centered representation until the motor error vector is reduced to zero. After such mismatch learning is complete, the auditory and motor vectors are calibrated to consistently represent target position in motor coordinates. This kind of vector mismatch learning is called *vector associative map*, or VAM, learning (Grossberg and Kuperstein, 1986, Chapter 4; Gaudiano and Grossberg, 1991). Such a transformation into motor coordinates can be learned, in parallel circuits, by any number of head-centered maps, including auditory, visually attentive, and planned movement maps.

VAM learning occurs only *after* an eye movement occurs, when the eyes maintain a steady posture, albeit one that may exhibit microsaccades. Movement gates enable learning to occur only at these times.

Suppose that learning has already occurred and a new target position is instated. *Before* a movement occurs, the new target position is read out at motor error vector cells, and the present eye position in motor coordinates is subtracted from it. This *difference vector* codes the desired movement direction and distance to the new target. In other words, the difference vector is a motoric representation of the motor error needed to move the eyes toward the desired target. Motor error vector cells hereby accomplish two things: they learn to transform a head-centered representation of a movement target location into motor coordinates, and, in so doing, they compute a difference vector that represents the direction and distance that the eyes need to move to foveate a new target. Otherwise expressed, the motor error vector processing stage accomplishes a learned coordinate change from a target's head-centered positional coordinates into retinotopically-consistent motor error coordinates. Moreover, this difference vector represents the same motor error as the position that the target activates on the SC motor error map.

### 10.5. Transforming a motor error vector into the SC motor error map

In order to transform, through learning, an auditory- or plan-derived motor error vector into the corresponding position on the SC motor error map, the model uses two stages of learning. The first learning stage converts a motor error vector into a motor error map. The second stage associates positions in this motor error map with the corresponding positions in the SC motor error map.

The first stage converts a motor error vector into a motor error map using a biologically-plausible instantiation of a self-organizing map, or SOM (e.g., Grossberg, 1976a, 1978; Grossberg and Kuperstein, 1986). This transformation is called *vector-to-map learning*. The usual instar gated steepest descent learning rule for SOMs is used (Grossberg, 1976a, 1980) whereby, when a motor error map cell is maximally activated and thereby wins the competition within the SOM, the activity of such a winning cell enables learning to occur within its abutting synapses from the motor error vector cells. The adaptive weight within each such synapse becomes proportional to the signal within its pathway from the corresponding motor error vector cell. In this way, learning maximizes the response of each winning motor error map cell. This vector-to-map learning process ensures that different motor error vectors get transformed into different motor error map positions.

The second learning stage associates the positions on the SOM with the corresponding motor error map positions in the deeper layers of the SC (Figure 5C).

These successive learned transformations were demonstrated through computer simulations in Grossberg et al. (1997). Their computational properties are consistent with the following anatomical refinement that brings these concepts into line with known SC and OT anatomy.

Figure 9 refines the Grossberg et al. (1997) proposal of how these two learning stages work (Figure 5C) by expanding the description, into separate excitatory and inhibitory multimodal nuclei, of how attentive ART matching and resonance helps to make positional choices that guide the map learning process, and dynamically stabilize it after learning occurs. This model variant enables the first vector-to-map learning stage to be compared with data concerning the parabigeminal nucleus in mammals and the isthmic nuclei in birds. The second map-to-map learning stage is interpreted to take place from these multimodal nuclei to the SC in mammals and the OT in birds.

Our analysis considers the case where the eyes can move in the head, even as the head moves in the body, to orient to movement targets, which is characteristic of mammals with a superior colliculus. However, some birds, such as barn owls, cannot move the eyes in the head by more than a few degrees (Knudsen, 1989). Some models have simplified the analysis of map learning by assuming that no eye movements at all occur in the barn owl, and that the eyes foveate straight ahead at all times in the head (e.g., Rucci et al., 1997). This simplifying assumption renders retinotopic and head-centered coordinates the same, since the eyes move rigidly with the head, but it does not deal with the issues that are raised by the more challenging case of eyes that can freely move in the head. Our analysis focuses on this more challenging case. Grossberg et al. (1997) discusses other properties that are missing from the Rucci et al. (1997) model.

### 10.6. Adaptive resonance, attention, choice, gamma oscillations, and normalization

In all self-organizing maps, the following basic question arises: How are stable learning and memory assured? As noted in Section 4, ART proposes how this happens using top-down attentive feedback. In the present case, a winning vector-to-map cell can activate a top-down, modulatory on-center, off-surround network that can focus attention upon the corresponding map position within the spreading wave layer of the SC (Figure 9). Topographically organized modulatory on-center, off-surround feedback from the spreading wave layer to the peak decay layer can also occur (Figure 9). These cooperative-competitive feedback loops trigger a synchronous resonant state between corresponding locations in motor error map, spreading wave, and peak decay cells. This attentive resonant state, which ART predicted can support synchronous oscillations, also called order-preserving limit cycles (Grossberg, 1976b, 1999), can be realized by gamma oscillations in the visual cortex (Grossberg and Versace, 2008) and the optic tectum (Knudsen, 2011; Sridharan et al., 2011). These gamma oscillations support the attentional focus upon the winning category representations and, at least in the SACCART model, drive self-stabilizing multimodal map learning by the corresponding synaptic weights.

In ART, the competitive interactions that control such attentional focusing are defined by recurrent on-center off-surround interactions between cells that obey membrane equation, or shunting dynamics. Such network interactions cause divisive normalization of cell activities (Grossberg, 1973, 1980). Such a divisive effect of lateral inhibition has been reported during feedback interactions between the OT and the Ipc in the owl (Asadollahi et al., 2011). Grossberg et al. (1997) also showed how such a recurrent shunting on-center off-surround network simulates the observed amplification of SC responses by positionally-convergent visual and auditory inputs, and their reduction by positionally-competing visual and auditory inputs (Stein and Meredith, 1993). As will be noted more completely below, the anatomical homolog of the model motor error map 1 is the Ipc and of the model motor error map 2 is the Imc.

A second source of inhibition comes from the basal ganglia (Hikosaka and Wurtz, 1983a,b), notably the substantia nigra pars reticulata (SNr). SNr inhibition gates the release of movement commands from the SC (Figures 6, 9). The burst and buildup cells cannot fire until the SNr inhibition is withdrawn. ART models of perception and cognition have proposed how opening of basal ganglia gates enables top-down modulatory on-center signals to become driving, and to thereby enable visual imagery, working memory storage, and internal thought to occur in different brain areas (Grossberg, 2000, 2013a; Grossberg and Pearson, 2008). In the case of movement control, opening these gates enables the release of chosen movement commands. Brown et al. (1999, 2004) and Silver et al. (2011) have developed the TELOS and lisTELOS models to quantitatively simulate neurophysiological and behavioral data about how the opening of basal ganglia movement gates learns to balance between the release of reactive and planned eye movements in a task-appropriate way.

### 10.7. Multimodal map learning implies spreading wave and peak decay cell properties

Map learning leads to a spread of activity across the buildup cell layer during saccades for the following reasons. A gaze motor error signal to the saccade generator (Figures 5A, 9) initiates an eye movement. As the eye moves, the corollary discharge of the changing eye position causes the motor error vector to decrease (Figure 5C). The decreasing error vector excites a series of positions on the motor error map. The motor error map then activates corresponding cell positions in the SC spreading wave, or buildup cell, layer. As the eye moves to foveate the target position, these SC positions represent movements closer to the fovea (Figures 5D, 9). During the eye movement, the sequence of activated positions of buildup cells hereby shifts across the SC map toward the foveal representation. Said in another way, the spreading wave is caused by continuous updating of the motor error map as the movement progresses. This view of buildup cell dynamics links spreading wave properties to the multimodal map learning process that enables auditory, visually attentive, planned, and visually reactive commands that represent the same position in space to maximally activate the same motor error map position within the SC.

How are peak decay cell properties related to this process? The spread of activity from its original location toward the fovea erodes feedback excitation to the burst cell map at which the visually reactive target was stored (Figure 9). This erosion is due to off-surround inhibition from the map position that represents the current eye position to the map position that represents the initial target position. This inhibitory feedback is part of the ART Matching Rule feedback loop that helps to dynamically stabilize the learned multimodal map. This explanation links the peak decay property to resonant multimodal map learning and motoric choice.

Each SC motor error map position codes the direction and amplitude (or length) of a saccadic movement. Such an encoding is needed to generate outflow movement commands, but it does not, in itself, accurately calibrate the ensuing saccades (e.g., see Stanford and Sparks, 1994; White et al., 1994; Stanford et al., 1996). Several other processes also need to be activated, and properly calibrated. For starters, the motor error map signal needs to converted to a temporal code that controls the firing rate of saccade generator cells (Robinson, 1973; Grossberg and Kuperstein, 1986, Chapter 7). There is a rich literature of models of how this conversion occurs. Gancarz and Grossberg (1998, 1999), for example, developed the FOVEATE model of the saccade generator, used it to quantitatively simulate psychophysical and neurophysiological data about saccadic eye movements, and compared FOVEATE with various other saccade generator models.

### 10.8. Cholinergically-modulated multimodal map learning in the avian optic tectum

The optic tectum (OT or TeO) plays a role in birds similar to that played by the SC in mammals. How well do the above results about multimodal map learning in the primate SC carry over to the avian OT (Knudsen and Brainard, 1995)? The nuclei isthmi pars parvocellularis (Ipc) and pars magnocellularis (Imc) are both reciprocally connected to the OT (Figure 10; Wang, 2003; Wang et al., 2004). The anatomy in Figure 10 of OT, Ipc, and Imc interactions is consistent with the SACCART model circuit in Figure 9, as well as those in Figures 5–8, which provide mechanistic explanations and predictions about the following types of data. Indeed, the predictions within the original Grossberg et al. (1997) SACCART article on attention, choice, and multimodal map learning in the SC have been strongly supported by subsequent data about the OT, as the following exposition will describe.

The Ipc connections with the OT are topographic and excitatory, whereas the Imc delivers broadly distributed inhibition to both the OT and Ipc (Marín et al., 2007), thereby together forming a recurrent on-center off-surround network that is capable of choosing the most salient target position (Grossberg, 1973; Koch and Ullman, 1985; Wang et al., 2004). In addition, the forebrain gaze control area (AGF), which is homologous to the mammalian frontal eye fields, also projects to the Ipc (Knudsen et al., 1995; Winkowski and Knudsen, 2008), and lesioning the AGF in behaving owls disrupts memory-guided saccades, much as a similar lesion to FEF affects behaving monkeys (Knudsen et al., 1995; Dias and Segraves, 1999).

Cells in the Ipc are multimodal (Maczko et al., 2006; Asadollahi et al., 2010), and AGF microstimulation modulates auditory as well as visual responses in the OT (Winkowski and Knudsen, 2008). AGF microstimulation sharpens auditory receptive fields at aligned OT positions, increasing their ability to resolve multiple sound stimuli while decreasing firing rates of cells at non-aligned OT positions. Changing inputs leads to a switch in the cells in Ipc and OT that fire briskly, and are consistent with the hypothesis that the recurrent Ipc on-center and Imc off-surround network helps to cause synchronized firing (i.e., resonance) across a broad domain of cells (Marín et al., 2007; Asadollahi et al., 2010). These and similar properties suggest that the Ipc plays a role in birds that may be functionally similar to the vector-to-map cells in the SACCART model, that the Imc supplies the off-surround in the on-center off-surround selection circuit, and that the reciprocal interactions of Ipc and Imc with OT are part of an adaptive resonance dynamic. In Figure 9, the motor error map 1 plays the role of the Ipc, whereas the motor error map 2 plays the role of the Imc.

It has been hypothesized that these feedback interactions help to create a winner-take-all circuit for the purpose of focusing spatial attention (Wang et al., 2004; Maczko et al., 2006) and lead to periodic bursts of spikes in the low gamma band (Asadollahi et al., 2010). This hypothesis for the OT is consistent with a similar SACCART hypothesis for the SC (see above, and Grossberg et al., 1997). Ipc neurons are cholinergic, and feedback from Ipc to OT initiates the bursting dynamics in the OT (Sorenson et al., 1989; Maczko et al., 2006; Marín et al., 2007) that exhibit the properties of an ART resonance.

The SACCART hypothesis predicts, in addition, how this circuitry, as a special case of ART dynamics, may control learning of the multimodal map that enables the OT and SC to focus spatial attention on the positions of chosen movement targets from other modalities than vision, notably audition and planned movement targets. Indeed, Marín et al (2012) have shown how synchronized feedback signals from the Ipc boost retinal signals to higher visual areas, hereby illustrating how the representations of movements to target positions and of the target cues themselves may be resonantly synchronized.

The hypothesis that SC and OT multimodal integration illustrates ART learning mechanisms is also consistent with evidence showing the importance of NMDA receptors for multimodal learning and integration in the deep layers of the cat superior colliculus (Schnupp et al., 1995; Binns and Salt, 1996; Huang and Pallas, 2001). Thus, the SC and OT seem to embody all the predicted ART linkages between processes of learning, expectation, attention, resonance, and synchrony.

### 10.9. Habituation and reset: is there SC mismatch reset and vigilance control?

Resonance brings with it the benefits of efficient neuronal processing and choice, as well as of self-stabilizing learning. However, because of the positive feedback loops in any resonance, there is also the risk of perseverative activation of winning cells. In a part of the brain's orienting system such as the SC and OT, it is particularly important that rapid changes in stimulus conditions lead to correspondingly rapid changes in saccadic targets to support successful survival. Neurophysiological data show that the SC and OT can, indeed, respond with rapid switching of their attended target locations (e.g., Goldberg and Wurtz, 1972; Ignashchenkova et al., 2003; Asadollahi et al., 2010). How does a flexible balance between resonance and reset occur? This is the type of question for which ART proposes two related solutions using novelty-sensitive mechanisms.

There are two different types of novelty mechanisms, but they share a key synaptic process. The first mechanism is the local habituative response that occurs whenever signals are gated by an activity-dependent habituative transmitter gate, or depressing synapse, as noted in Section 4.6. Previously habituated positions are at a competitive disadvantage during the bottom-up selection of a newly stimulated target position. Such activity-dependent habituation, or medium term memory (Grossberg, 1969, 1972, 2013a,b; Francis et al., 1994; Francis and Grossberg, 1996; Grossberg and Versace, 2008), is one of the mechanisms that contributes to flexible reset. One way that SC neurons can distinguish between novel and persistent stimuli is thus by habituating in an activity-dependent way after an initially strong response to a visual stimulus (Oyster and Takahashi, 1975; Stein, 1984; Sparks and Nelson, 1987; Cirone and Salt, 2001; Boehnke et al., 2011). Indeed, neurons in the SC respond preferentially to the sudden onset of a novel or behavioral significant stimulus and generate appropriate behavioral and avoidance responses to these events (Sparks and Nelson, 1987; Cirone and Salt, 2001). Perrault et al. (2011) noted, in addition, that SC responses that are initially weak tend to potentiate, whereas responses that are initially strong tend to habituate, and a subset of active neurons responded to a novel event with dishabituation late in the visual response profile for both brighter and dimmer stimuli (Boehnke et al., 2011).

The second kind of novelty mechanism is the mismatch-mediated kind of novelty response that occurs when a currently active top-down expectation mismatches a bottom-up input pattern. Such a mismatch can trigger a burst of activation that is non-specifically broadcast across multiple brain areas. When such an arousal burst is gated by habituative synapses, it can rapidly reset ongoing activity to enable a response appropriate to the novel stimulus to rapidly take hold (Figure 3; Grossberg, 1972, 1984c). This mismatch-mediated type of reset is what vigilance control regulates (Sections 1, 2, and 5). Moreover, the level of vigilance, and thus the conditions under which mismatch-mediated reset occurs, has been hypothesized to be influenced by acetylcholine (Section 9).

Is there experimental data to support the hypothesis that cholinergically-modulated mismatch-mediated arousal bursts help to reset attended target positions in the SC and OT? The following experimental facts lead credence to this hypothesis, which needs further testing to be fully supported or disconfirmed: The pedunculopontine tegmental nucleus (PPTN), a part of the brain's ascending activating system, is a brainstem cholinergic nucleus, and facilitates generation of SC motor outputs for the initiation of saccades (Krauthamer et al., 1995; Kobayashi and Isa, 2002). In addition, the SC projects to the intralaminar thalamus, which in turn projects to motor cortex and the basal ganglia, and is part of the brain's arousal system (Grunwerg and Krauthamer, 1992).

One way to further test this hypothesis is to study the timing of the reset of a visually-activated target location in the deeper layers of the SC or OT in response to a sudden, unexpected, and loud auditory stimuli from a different location in space. Is there an arousal component to the reset of the visually-activated target location, or is it entirely driven by competition within the SC by the incoming auditory signal? If the former is true, then it will be of great interest to test whether there is a homolog in the SC and OT of the kind of nucleus basalis regulation of vigilance and reset that goes on in the neocortex.

## 11. Concluding remarks

The current article describes how feedback interactions in both cognitive and motor circuits can regulate resonant attention and choice, which in turn can trigger self-stabilizing category learning. The cognitive categories learn to recognize objects in the world. The motor categories learn a multimodal map for the selection of saccadic eye movement targets. Both types of circuits seems to share similar features that are needed to achieve a flexible balance between resonance and reset, where resonance focuses attention and drives learning of categories and expectations, whereas reset enables a flexible shift of attention in response to an unexpected event toward the objects and positions that are needed to deal with this event. Both types of circuits use habituative synapses and cholinergically-modulated interactions to achieve the resonance-reset balance. Accumulating evidence supports the prediction that cholingerically-mediated vigilance control can modulate the concreteness of cognitive category attention and learning. The nucleus basalis seems to play an important role in this type of mismatch-mediated reset. Although available data are consistent with the possibility that a similar type of modulation occurs in the SC and OT, further experiments are needed to clarify whether this homology is complete, experiments that can profitably exploit the multimodal convergence of conflicting signals from both visual and auditory cues in the deeper layers of the superior colliculus.

### Conflict of interest statement

The authors declare that the research was conducted in the absence of any commercial or financial relationships that could be construed as a potential conflict of interest.
